# The biogeochemical vertical structure renders a meromictic volcanic lake a trap for geogenic CO_2_ (Lake Averno, Italy)

**DOI:** 10.1371/journal.pone.0193914

**Published:** 2018-03-06

**Authors:** Franco Tassi, Stefano Fazi, Simona Rossetti, Paolo Pratesi, Marco Ceccotti, Jacopo Cabassi, Francesco Capecchiacci, Stefania Venturi, Orlando Vaselli

**Affiliations:** 1 Department of Earth Sciences, University of Florence, Via G. La Pira 4, Florence, Italy; 2 IGG-CNR Institute of Geosciences and Earth Resources, National Research Council of Italy, Via La Pira 4, Florence, Italy; 3 IRSA-CNR Water Research Institute, National Research Council of Italy, Via Salaria, Monterotondo, Rome, Italy; Tampere University of Technology, FINLAND

## Abstract

Volcanic lakes are characterized by physicochemical favorable conditions for the development of reservoirs of C-bearing greenhouse gases that can be dispersed to air during occasional rollover events. By combining a microbiological and geochemical approach, we showed that the chemistry of the CO_2_- and CH_4_-rich gas reservoir hosted within the meromictic Lake Averno (Campi Flegrei, southern Italy) are related to the microbial niche differentiation along the vertical water column. The simultaneous occurrence of diverse functional groups of microbes operating under different conditions suggests that these habitats harbor complex microbial consortia that impact on the production and consumption of greenhouse gases. In the epilimnion, the activity of aerobic methanotrophic bacteria and photosynthetic biota, together with CO_2_ dissolution at relatively high pH, enhanced CO_2_- and CH_4_ consumption, which also occurred in the hypolimnion. Moreover, results from computations carried out to evaluate the dependence of the lake stability on the CO_2_/CH_4_ ratios, suggested that the water density vertical gradient was mainly controlled by salinity and temperature, whereas the effect of dissolved gases was minor, excepting if extremely high increases of CH_4_ are admitted. Therefore, biological processes, controlling the composition of CO_2_ and CH_4_, contributed to stabilize the lake stratification of the lake. Overall, Lake Averno, and supposedly the numerous worldwide distributed volcanic lakes having similar features (namely bio-activity lakes), acts as a sink for the CO_2_ supplied from the hydrothermal/magmatic system, displaying a significant influence on the local carbon budget.

## Introduction

The occurrence of a lake in active and quiescent volcanoes is a common feature, as demonstrated by the 474 volcanic lakes listed in the VOLADA database (https://vhub.org/tags/voladadatabase). Dissolved gases in volcanic lake environments have a multiple origin: (i) deep fluid sources, i.e. magma degassing and hydrothermal fluid-rock interactions, (ii) atmospheric gases dissolved in ground and lake water and (iii) metabolic processes of living organisms [1]. Lakes hosted in quiescent volcanoes receive only minor amounts of heat and strong acidic gases (e.g. SO_2_ and HCl) from the hosting system, the deep fluid source mainly consisting of almost pure CO_2_ [2]. Hence, these volcanic lakes are characterized by relatively low water temperature and salinity, neutral to slightly acidic pH and a permanent thermal and chemical stratification [3]. Such physicochemical conditions are particularly favorable for the development of a CO_2_-rich gas reservoir [4], where occasional rollover events can cause the poisoning of shallow water layers and the release of killing gases into the atmosphere (the so-called limnic eruptions; [5].

The physicochemical mechanisms regulating the occurrence of gas bursts from volcanic lakes were intensively studied after the two disasters occurred in 1984 and 1986 at Nyos and Monoun lakes (Cameroon), respectively [6–14]. Intervention plans aimed to mitigate this natural hazard were strongly debated ([15–18]. At Lake Nyos, the amount of dissolved CO_2_ in the hypolimnion, i.e. the main risk factor for limnic eruptions, was decreased by installing 3 pipelines into the gas reservoir to spontaneously allow degassing the lake [19–22]. However, such a drastic intervention causes a direct release of undesired greenhouse gases into the atmosphere and may significantly affect the delicate lake ecosystem [23]. The application of similar remediation has to be carefully evaluated.

**Fig 1:**
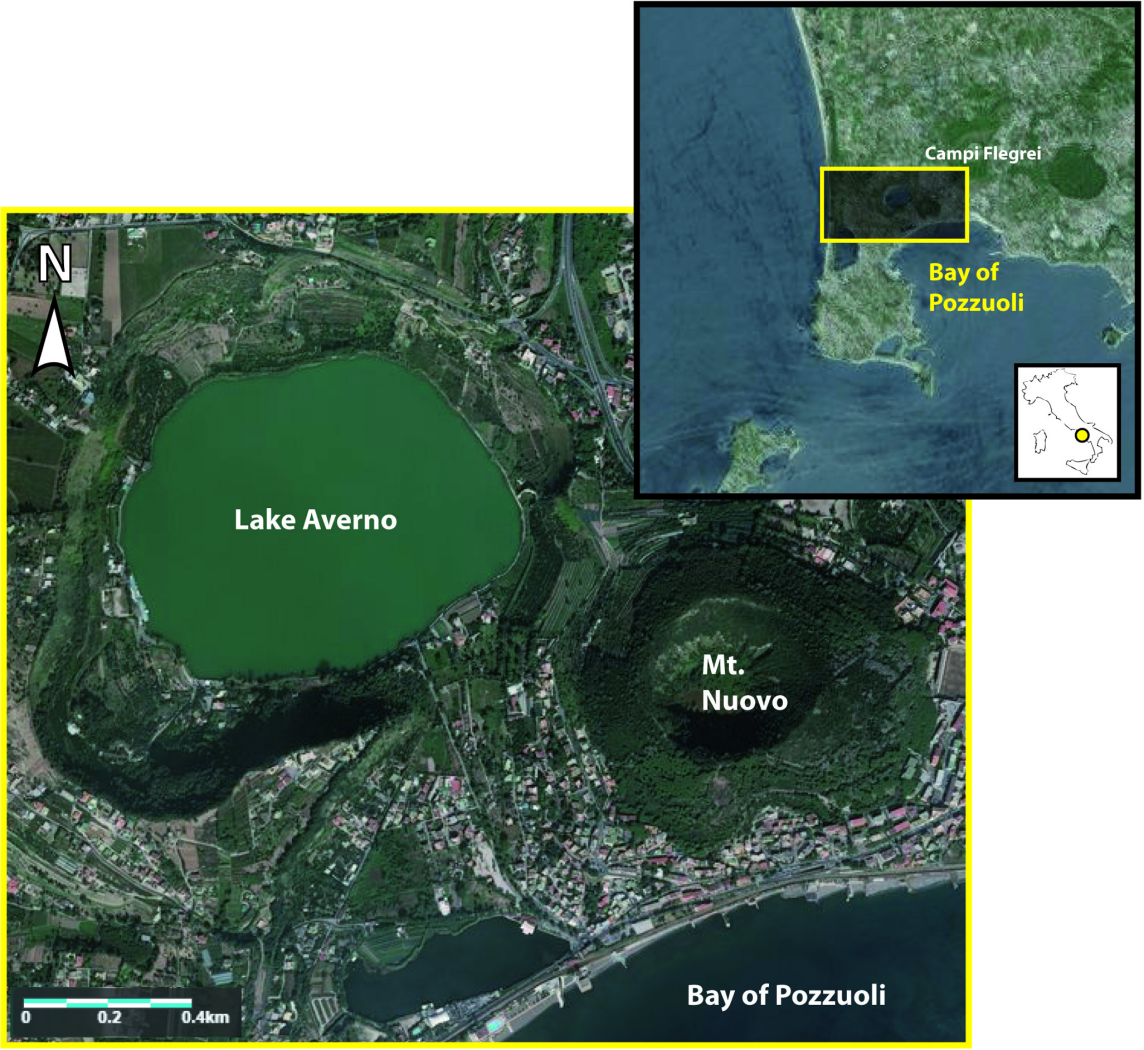
Schematic map of the Campi Flegrei caldera with the location of Lake Averno. This Figure is similar but not identical to the original image, and is therefore for illustrative purposes only.

Meromictic volcanic lakes characterized by a relatively low input rate of volcanic-hydrothermal gases (CO_2_) typically show dissolved CH_4_, produced by microbial activity occurring in the bottom sediments and within the water column, at concentrations comparable to those of CO_2_ [24–28]. External gas (CO_2_) inputs play as a trigger for prokaryotic activity [29–31], whose activity along the water column and in the bottom sediments likely represents the main controlling factor for the development and temporal evolution of a dissolved gas reservoir within these lakes. According to these considerations, they were classified as *bio-activity* volcanic lakes [32]. In these systems, the vertical distribution of bacterioplankton is closely related to the vertical gradient of the physicochemical features, as postulated by Paganin et al. [33] in a recent study on Lake Averno (Campi Flegrei, southern Italy; Fig 1), a maar lake generated while two eruptions occurred 3.7–4.5 ky BP [34]. In this lake, fish kill events accompanied by evident color changes of the lake surface related to the Fe^2+^ oxidation, were occasionally observed in winter periods, as a consequence of water overturn from top (epilimnion) to bottom (hypolimnion) possibly due to a strong temperature decrease (<7°C) of the surficial waters [26]. It is worth noting that *Averno* derives from *Aornon* a Greek word meaning “without birds”, likely related to the occurrence within the lake basin of volcanic gas exhalations. Water overturns were also reported for other meromictic volcanic lakes in Italy, such as Lake Albano, nearby the city of Rome [35], and Lake Piccolo, nested in a crater of Mt. Vulture volcano in the Basilicata Region [36–37]. Overall, the complex interplay between (i) the dynamics of microbial populations and (ii) the chemistry of water and dissolved gases in bio-activity lakes can properly be investigated only by combining a microbiological and geochemical approach.

The main aims of the present study were to (i) unveil the microbial-driven chemical reactions governing the chemistry of Lake Averno, with a special focus on the main dissolved gases (CH_4_ and CO_2_), and (ii) evaluate the influence of these bio-geochemical processes producing and consuming the main dissolved gases on the mechanisms regulating the development of the gas reservoir and the stability of the lake stratification. For these goals, the chemical and isotopic composition of waters and dissolved gases measured along the Lake Averno water column was coupled with the determination of vertical distribution and diversity of prokaryotes. Bacterial and archaeal assemblages were assessed at different levels of phylogenetic resolution by determining (i) abundance of prokaryotes and community composition at single cell level by fluorescence in situ Hybridization Catalyzed Reported Deposition (CARD-FISH) and (ii) microbial diversity using next generation sequencing (NGS).

## Materials and methods

### Ethics statement

The data were analyzed anonymously.

The field studies did not involve endangered or protected species.

No permissions were required to access the study area, which is a Lake that can be accessed by population.

### Field measurements

Water temperature, dissolved O_2_, electrical conductivity (EC) and pH were measured on 17^th^ June 2015 using a multi-parametric probe (Hydrolab IP188A multi-probe) equipped with a data logger for data storage. The probe, whose data acquisition frequency was 5 s, was very slowly lowered from the surface to the maximum lake depth (34 m) to obtain measurement intervals <15 cm. The nominal precisions were as follows: depth ±0.05 m; temperature ±0.03°C; pH ±0.1, O_2_ ±1.56 μmol/L; EC ±0.01 mS/cm. Alkalinity (alk) was measured by acidimetric titration (AC) with 0.01 N HCl using a Metrohm 794 automatic titration unit. The analytical error for AC analysis was ≤5%.

### Water and dissolved gas sampling

The sampling of water and dissolved gases for both the geochemical and microbiological analyses, as well as the field measurements, were carried out just after the multi-parametric probe measurements along a vertical profile from the lake surface to the maximum depth (34 m), at regular intervals of 2 m. According to the single hose method [4], a small diameter (6 mm) Rilsan tube, lowered at the sampling depth and connected to a 100 mL syringe equipped with a three-way Teflon valve, was used to pump up the water. After the displacement of a water volume at least twice the inner volume of the tube, one filtered (0.45 μm) and two filtered–acidified (with ultrapure HCl and HNO_3_, respectively) water samples were collected in polyethylene bottles for the analysis of anions, cations, and trace species, respectively. Reduced sulfur species (expressed as ΣS^2-^ and mainly consisting of H_2_S, HS^-^, S^2-^) were analyzed on 8 mL water samples collected in 15 mL plastic tubes after the addition of 2 mL of a Cd-NH_4_ solution (Cd-IC method; [38]).

The isotope analyses of water (δD-H_2_O and δ^18^O-H_2_O) and total dissolved inorganic carbon (δ^13^C-TDIC) were carried out on samples collected in 40 mL glass bottles with the addition of few milligrams of HgCl_2_ to prevent any fractionation process of carbon isotopes due to the presence of bacterial activity [39]. Samples for concentrations and isotopic analyses of dissolved gases were collected using pre-evacuated 250 mL glass vials equipped with a Teflon stopcock. Once the vial was connected to the Rilsan tube through the three-way valve, the stopcock was opened to allow water entering up to about three fourths of the vial inner volume [39].

For the analysis of microbial diversity, lake water (250 mL) was collected at 8 depths (0 m; -6 m; -16 m; -20 m; -24 m; -28 m; -32 m; -34 m), filtered on-deck using a Millipore Sterivex filter unit (pore size 0.22 μm) and immediately stored on-deck in dry ice. For the analysis of community composition by CARD-FISH, a further aliquot of water (500 mL) was fixed for 2 h at 4°C with formaldehyde solution (37% w/v, Sigma Aldrich; final concentration 1%) (Fazi et al. [40–41]). Sub-aliquots of 5–10 mL were filtered at low vacuum levels (<0.2 bar) onto 0.2 μm pore-size polycarbonate filters (type GTTP; diameter, 47 mm; Millipore, Eschborn, Germany). Sterivex filter units and CARD-FISH filters were stored at -20°C until further processing.

### Chemical, isotopic and microbiological analyses

#### Waters

The main anions (Cl^-^, SO_4_^2-^, NO_3_^-^, Br^-^, and F^-^) and cations (Na^+^, K^+^, Ca^2+^, Mg^2+^, NH_4_^+^ and Li^+^) were analyzed by ion chromatography (IC) using Metrohm 761 and Metrohm 861 chromatographs, respectively. Reduced sulfur species (ΣS^2-^) were analyzed as SO_4_^2-^ by IC (Metrohm 761) after oxidation with H_2_O_2_ of CdS formed by the reaction between ΣS^2-^ and the Cd-NH_4_ solution [38]. Trace elements (P, Fe_tot_, Mn and Zn) were analyzed by inductively coupled plasma optical emission spectrometry (ICP-OES) using a Perkin Elmer Optima 8000. The analytical errors for IC and ICP-OES analysis were <5% and <10%, respectively.

The D/^1^H and ^18^O/^16^O ratios of water (expressed as δD-H_2_O and δ^18^O-H_2_O in ‰ vs. V-SMOW) were determined using a Finnigan Delta Plus XL mass spectrometer according to standard protocols. Oxygen isotopes were analyzed using the CO_2_–H_2_O equilibration method [42]. Hydrogen isotopes were analyzed on H_2_ produced after the reaction of 10 mL of water with metallic zinc at 500°C. Analytical errors for δD-H_2_O and δ^18^O-H_2_O analysis were ±0.1‰ and ±1‰, respectively.

The ^13^C/^12^C ratios of Total Inorganic Carbon (expressed as δ^13^C-TDIC in ‰ vs. V-PDB) were determined on CO_2_ produced by reaction of 3 mL of water with 2 mL of anhydrous phosphoric acid *in vacuum* [43–44] by mass spectrometry (MS) using a Finnigan Delta Plus XL, after two-step extraction and purification procedures of the gas mixtures by using liquid N_2_ and a solid–liquid mixture of liquid N_2_ and trichloroethylene [45]. Internal (Carrara and San Vincenzo marbles) and International (NBS18 and NBS19) standards were used for estimating the external precision. The analytical error and the reproducibility for MS analysis were ±0.05‰ and ±0.1‰, respectively.

#### Dissolved gases

The compositions of the inorganic dissolved gases in the headspace of the sampling flasks (CO_2_, N_2_, Ar+O_2_, H_2_ and He) was determined by gas chromatography (GC) using a Shimadzu 15A equipped with a 5 m long stainless steel column packed with Porapak 80/100 mesh and a Thermal Conductivity Detector (TCD), whereas CH_4_ was analyzed using a Shimadzu 14A equipped with a 10 m long stainless steel column packed with Chromosorb PAW 80/100 mesh coated with 23% SP 1700 and a Flame Ionization Detector (FID) [45–46]. Argon and O_2_ were analyzed using a Thermo Focus gas chromatograph equipped with a 30 m long capillary molecular sieve column and a TCD. The analytical error for GC analysis was ≤5%. Assuming that in the sampling flasks the separated gas phase was in equilibrium with the liquid, the number of moles of each gas species in the liquid (n_i,l_) was calculated on the basis of those in the flask headspace (n_i,g_) by means of the Henry's law constants [47]. The total moles of each gas species in the water sample was given as the sum of n_i,l_ and n_i,g_. The partial pressures of each gas species were then computed, based on the total mole values according to the ideal gas law.

The isotopic composition of dissolved CO_2_ (δ^13^C-CO_2_ expressed as ‰ vs. V-PDB) was determined by analyzing the ^13^C/^12^C ratio of CO_2_ in the sampling flask headspace (δ^13^C_CO2meas_) using the same instrument and purification procedure used for the determination of the δ^13^C-TDIC values. The δ^13^C values of dissolved CO_2_ were then calculated from the measured δ^13^C_CO2meas_ on the basis of the enrichment factor (ε_1_) for gas-water isotope equilibrium [48], as follows:

$$\varepsilon_{1}=\delta^{13}C-CO_{2}-\delta^{13}C-CO_{2meas}=\left(0.0049\times T\right)-1.31$$ (1)

where temperature (T) is expressed in °C. The analytical error and the reproducibility for δ^13^C-CO_2_ analysis were ±0.05‰ and ±0.1‰, respectively.

The analysis of the ^13^C/^12^C and D/^1^H ratios of CH_4_ (δ^13^C-CH_4_ and δD-CH_4_, expressed as ‰ vs. V-PDB and ‰ vs. V-SMOW, respectively) was carried out by MS using a Varian MAT 250 according to the procedure and the sample preparation described by Schoell [49]. The analytical error for δ^13^C-CH_4_ and δD-CH_4_ analysis was ±0.15%.

#### Prokaryotic abundance and community composition by fluorescence in situ hybridization analysis

Filter sections were stained with DAPI at a final concentration of 1 μg/mL to quantify the total Prokaryotes. At least 20 microscopic fields were counted, including a minimum of 800 DAPI-stained cells. Photosynthetic picoplankton (Cyanobacteria) were discriminated for the reddish autofluorescence (excitation wavelength 550 nm; CY3).

Community composition was assessed by CARD-FISH following the protocol optimized by Fazi et al. [40–41]. rRNA-target Horseradish peroxidase (HRP) labeled oligonucleotidic probes (Biomers, Ulm, Germany) were used to target Bacteria (EUB338 I-III), and Archaea (ARCH915). Moreover, the following HRP-labelled probes were used: ALF968, targeting sequence types affiliated with Alphaproteobacteria; BET42a for Betaproteobacteria; GAM42a for Gammaproteobacteria; DEL 495 a-b-c for Deltaproteobacteria; EPSY 914 and EPSY 549 for Epsilonproteobacteria; CF319a for Bacteroidetes (formerly Cytophaga-Flavobacterium-Bacteroides) [50]. The stained filter sections were inspected on a Leica DM LB 30 epifluorescence microscope (Leica Microsystems GmbH, Wetzlar, Germany) at 1000x magnification. At least 300 cells were counted in >10 microscopic fields randomly selected across the filter sections. The relative abundance of hybridized cells was estimated as the ratio of hybridized cells to total DAPI-stained cells.

#### Bacterial diversity by next generation sequencing

DNA was extracted from Sterivex filter units using the PowerSoil^®^ DNA Isolation Kit (MoBio Laboratories Inc., California) as described for water samples by Chandler et al. [51]. The manufacturer’s instructions were followed, although minimal modifications were applied with the aim of increasing the DNA quality and yield [52]. The concentration of each DNA extract was quantified by Nanodrop (Thermos Scientific), and the DNA stored at −80°C until further processing.

V3-4 16S rRNA gene sequencing libraries were prepared by a custom protocol based on an Illumina protocol [53] using 10 ng of extracted DNA as template for PCR amplification of 16S rRNA gene fragments. Each PCR reaction (25 μL) contained dNTPs (400 μM of each), MgSO_4_ (1.5 mM), Platinum^®^ Taq DNA polymerase HF (0.5 U), 1X Platinum^®^ High Fidelity buffer (Thermo Fisher Scientific, USA) and tailed primermix (400 nM of each forward and reverse). PCR was run with following program: Initial denaturation at 95°C for 2 min, 35 cycles of amplification (95°C for 20 s, 50°C for 30 s, 72°C for 60 s) and a final elongation at 72°C for 5 min. Duplicate PCR reactions were performed for each sample and the duplicates were pooled after PCR. The forward and reverse primers utilized were: Bacteria/Archaea V3-4 5’-CCTAYGGGRBGCASCAG (341F) and 5’-GGACTACNNGGGTATCTAAT (806R) [54]. The amplicon libraries were purified using Agencourt Ampure XP Bead (Beckman Coulter, USA), following the vendor recommended protocol, using a sample:bead ratio of 5:4, and the DNA was eluted in 33 μL of nuclease free water (Qiagen, Germany). DNA concentration was measured using Quant-iT DNA Assay Kit, high sensitivity (Thermo Fisher Scientific, USA).

Sequencing libraries were prepared from the purified amplicon libraries using a second PCR. The sequencing libraries were purified using Agencourt Ampure XP Bead (Beckman Coulter, USA) following the vendor recommended protocol, using a sample:bead ratio of 5:4, and the DNA was eluted in 20 μL of nuclease free water (Qiagen, Germany). DNA concentration was measured using Quant-iT DNA Assay Kit, high sensitivity (Thermo Fisher Scientific, USA). Gel electrophoresis using Tapestation 2200 and D1000 High Sensitivity screentapes (Agilent, USA) was used to check the product size and purity of randomly picked sequencing libraries.

The purified sequencing libraries were pooled in equimolar concentrations and diluted to 4 nM. The samples were paired end sequenced (2×301bp) on a MiSeq (Illumina) using a MiSeq Reagent kit v3, 600 cycles (Illumina) following the standard guidelines for preparing and loading samples on the MiSeq. 10%Phix control library was spiked in to overcome low complexity issue often observed with amplicon samples.

For 16S rRNA amplicon bioinformatic processing, the generic workflow reported in Karst et al. 2016 [55], was followed. V3-4 forward and reverse reads were trimmed for quality using Trimmomatic v. 0.32 [56] with the settings SLIDINGWINDOW:5:3 and MINLEN:275. The trimmed forward and reverse reads were merged using FLASH v. 1.2.7 [57] with the settings -m 25 -M 200. The merged reads were dereplicated and formatted for use in the UPARSE workflow [58]. The dereplicated reads were clustered, using the usearch v. 7.0.1090 -cluster_otus command with default settings. Operational taxonomic units (OTUs) abundances were estimated using the usearch v. 7.0.1090 –usearchglobal command with -id 0.97. Taxonomy was assigned using the RDP classifier [59] as implemented in the parallel_assign_taxonomy_rdp.py script in QIIME [60], using the updated version of the SILVA. The similarity-level used in OTU classification was 97% (default option for the cluster_otus command). Chimeras were removed as default option. All sequences were deposited in the NCBI SRA database under the BioProject ID PRJNA421290 (SRP126315).

### Data analysis

NGS results were analyzed in R (R Core Team 2015) through the Rstudio IDE using the ampvis package v.1.9.1 [61]. Data in the OTU table were normalized to percent. Moreover, in order to calculate to calculate alpha-diversity the data were normalized to the same number of reads (subsample of 1500 reads). Data were also presented as the 40 most abundant bacterial genera in the samples. Eventually, the abundances of each genus were normalized with respect to the average abundance. Hence, this allowed visualization of the relative increase or decrease in abundance of each *genus* at the 8 sampling depths, despite the differences in abundance between genera. In addition, the genera were clustered (the y-axis) to visualize those with similar patterns. Clustering was conducted using the hclust command implemented in R using default settings with the percentage abundances.

A Nonmetric MultiDimensional Scaling ordination plot (NMDS), based on the Bray-Curtis dissimilarity matrix, was used to graphically visualize microbiological taxonomic composition (40 most abundant genera) in the samples across sites. The major chemical characteristics (HCO_3_^-^, F^-^, Cl^-^, Br^-^, P, NO_3_^-^, SO_4_^2-^, Ca^2+^, Mg^2+^, Na^+^, K^+^, NH_4_^+^, Fe, Mn, Zn, Li^+^, TDS) and dissolved gases (CO_2_, N_2_, Ar, CH_4_, O_2_, H_2_, He) were incorporated in the analysis with a vector-fitting procedure. The correlation coefficients between each environmental variable and the NMDS scores were presented as vectors from the origin with the length scaled to make a readable biplot. Stress value indicates the significant concordance between the distance among samples in the NMDS plot and the actual Bray-Curtis distance among samples [62].

## Results

### Water temperature, EC, pH and dissolved O_2_

**Fig 2:**
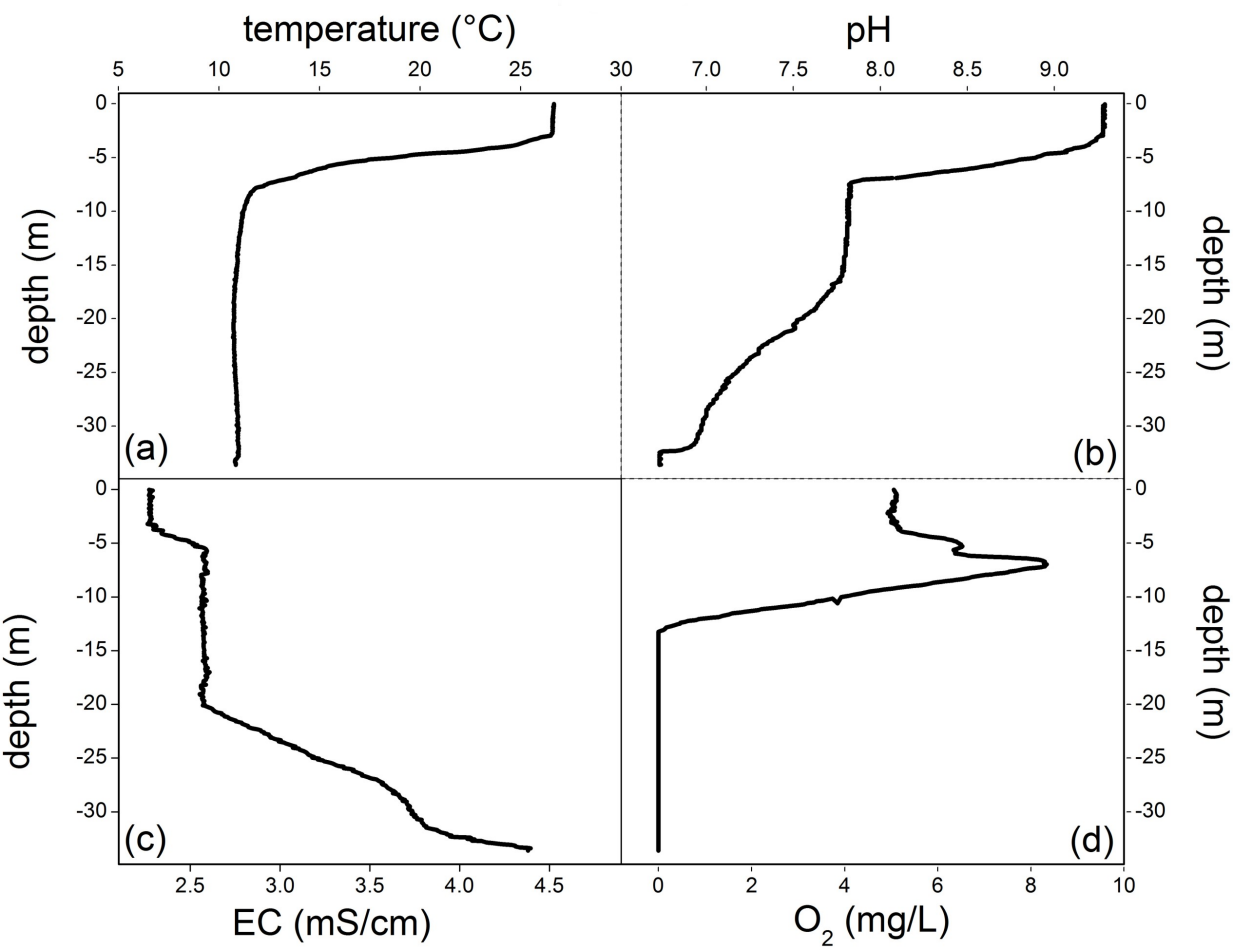
Vertical profiles along the Lake Averno water column of (a) water temperature (°C), (b) pH, (c) electrical conductivity (EC, in mS/cm), and (d) dissolved O_2_ (mg/L).

As shown in Fig 2A, a strong thermocline occurred at 3–7 m depth, separating a relatively warm (up to 26.6°C) epilimnion from a cold hypolimnion, the latter being at 12°C. The pH values followed a similar vertical pattern (Fig 2B), being characterized by relatively high values (>9) at depth ≤4 m, a sharp decrease from 4 to 7 m depth and a less pronounced decrease down to the bottom layer, where the minimum value (6.73) was measured. The vertical distribution of the EC values was marked by three haloclines (Fig 2C), the shallowest one in correspondence of the thermocline, the main one at 20–28 m depth, and the third one at >32 m depth, where a strong EC increase was also measured in the past surveys. Dissolved O_2_ had a clinograde profile in correspondence with the thermocline (Fig 2D), i.e. a strong decrease with depth typical of meromictic lakes. The positive heterograde oxygen curve occurring in the metalimnion, i.e. an O_2_ increase with depth commonly ascribed to oxygen production from algae populations favored by the large availability of nutrients [63], contrasts with the relatively low dissolved O_2_ concentrations in the epilimnion, which were lower than those expected at saturation at the measured water temperature.

### Chemical and isotopic composition of water

**Fig 3:**
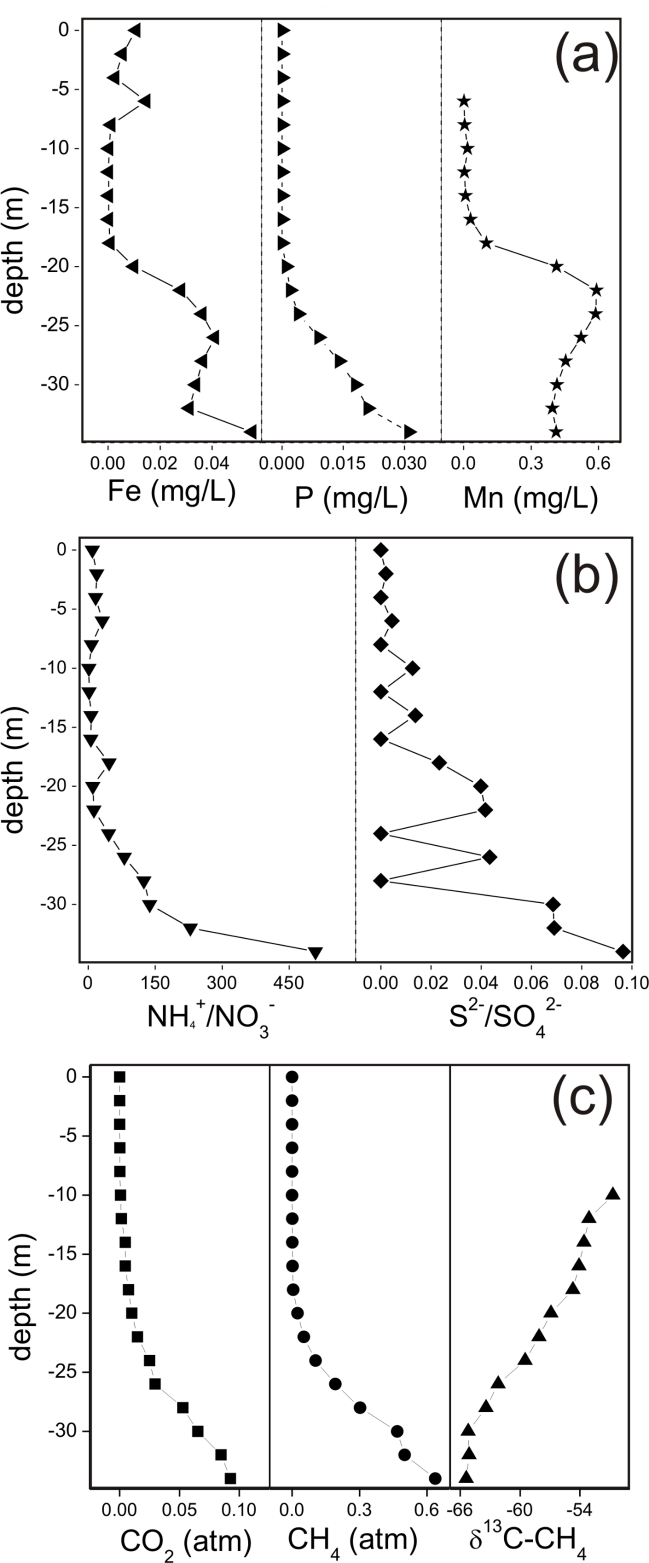
Vertical profiles along the Lake Averno water column of (a) P, Fe and Mn concentrations, (b) ΣS^2-^/SO_4_^2-^ and NH_4_^+^/NO_3_^-^ ratios, and (c) δ^13^C-CH_4_ values (‰ vs. V-PDB) and CO_2_ and CH_4_ partial pressures (atm).

Lake Averno showed relatively high values of total dissolved solids (TDS), which were relatively constant (1,739–1,779 mg/L) at depth < 20 m and increased up to 2,805 mg/L in the deepest water layer. The chemical composition was dominated by Na^+^ and Cl^-^, whose concentrations were up to 645 and 783 mg/L, respectively, with relatively high values of alkalinity (up to 944 mg/L) and SO_4_^2-^ (up to 200 mg/L) (Table 1). Significant concentrations of Ca^2+^ (up to 83 mg/L), K^+^ (up to 72 mg/L), NH_4_^+^ (up to 36 mg/L) and Mg^2+^ up to 27 mg/L) were also measured. Lower concentrations were measured for F^-^, Br^-^, NO_3_^-^ and Li^+^ (up to 13, 2.2, 1.2 and 0.85 mg/L, respectively), whilst Fe_tot_ and Zn were ≤0.055 and ≤0.0063 mg/L, respectively. Detectable concentrations (>0.0005 mg/L) of Mn (up to 0.41 mg/L) and P (up to 0.031 mg/L) were measured at depths ≥6 and ≥20 m, respectively (Fig 3A), whereas the concentrations of ΣS^2-^, measured at selected depths (Table 1), ranged from 0.41 to 18 mg/L. Both the NH_4_^+^/NO_3_^-^ and the ΣS^2-^/ SO_4_^2-^ ratios significantly increased with depth (up to 509 and 0.096, respectively) (Fig 3B).

**Table 1:** Sampling depth (m), temperature (°C), pH, salinity (expressed as TDS, in mg/L) alkalinity (alk, in mg/L) and chemical composition (in mg/L) of the main solutes, reduced sulfur species and trace elements in water samples from Lake Averno. The δ^13^C-TDIC (in ‰ vs. V-PDB), δD-H_2_O (in ‰ vs. V-SMOW) and δ^18^O-H_2_O (in ‰ vs. V-SMOW) values are also reported.

depth	T	pH	alk	F^-^	Cl^-^	Br^-^	P	NO_3_^-^	SO_4_^2-^	Ca^2+^	Mg^2+^	Na^+^	K^+^
0	26.6	9.29	403	10.7	549	1.5	<0.0005	0.24	200	18	16	512	65
2	26.6	9.28	395	9.8	546	1.5	<0.0005	0.12	202	17	15	521	66
4	24.3	9.14	413	10.1	537	1.5	<0.0005	0.11	197	17	12	502	68
6	15.0	8.53	417	9.89	535	1.5	<0.0005	0.072	195	24	13	484	57
8	11.7	7.83	427	10.6	540	1.5	<0.0005	0.24	194	26	12	483	51
10	11.2	7.82	429	10.5	548	1.5	<0.0005	1.1	194	30	14	483	55
12	11.3	7.81	419	9.64	536	1.6	<0.0005	1.2	195	29	13	490	50
14	10.9	7.80	419	10.3	542	1.6	<0.0005	0.38	197	31	13	474	53
16	10.8	7.78	416	10.5	539	2.0	<0.0005	0.27	195	30	13	479	52
18	10.7	7.67	415	10.1	541	1.5	<0.0005	0.082	195	32	14	482	52
20	10.7	7.53	411	10.0	550	1.6	0.001	0.38	197	32	14	485	55
22	10.8	7.38	426	9.94	567	1.5	0.002	0.35	199	36	14	499	56
24	10.8	7.23	460	10.1	592	1.6	0.004	0.17	203	40	17	510	57
26	10.9	7.12	586	9.74	632	1.8	0.009	0.16	195	49	18	550	57
28	10.9	7.02	676	9.90	680	1.8	0.014	0.13	197	59	19	576	62
30	10.9	6.97	761	10.0	706	2.0	0.018	0.16	191	63	20	592	64
32	11.0	6.89	814	10.2	730	2.1	0.021	0.11	191	70	22	622	66
34	10.8	6.73	944	10.7	783	2.2	0.031	0.07	185	83	27	645	72
depth	NH_4_^+^	Li^+^	ΣS^2-^	Fe_tot_	Mn	Zn	TDS	δ^13^C-TDIC_calc_	δ^13^C-TDIC	δD-H_2_O	δ^18^O-H_2_O		
0	2.2	0.72		0.011	<0.0005	0.0063	1,779		-2.91	-13.08	-1.35		
2	2.3	0.76	0.41	0.0056	<0.0005	0.0062	1,777		-2.78	-12.58	-1.28		
4	1.9	0.61		0.0027	<0.0005	0.0028	1,761		-2.95	-12.07	-1.07		
6	2.3	0.56	0.87	0.015	0.0011	0.0030	1,740	-2.9	-3.02	-12.55	-1.05		
8	1.7	0.55		0.0012	0.0045	0.012	1,748	-2.9	-3.11	-12.71	-1.14		
10	1.5	0.48	2.5	0.00031	0.017	0.0022	1,770	-3.8	-3.56	-12.39	-1.15		
12	2.3	0.58		0.00029	0.0036	0.0013	1,747	-3.4	-4.11	-12.11	-1.11		
14	2.4	0.72	2.7	0.00025	0.0084	0.0017	1,747	-2.8	-4.39	-11.54	-1.05		
16	1.6	0.69		0.00027	0.031	0.0013	1,739	-3.0	-4.87	-11.89	-1.19		
18	3.8	0.47	4.6	0.00083	0.10	0.0014	1,753	-1.7	-5.06	-12.41	-1.12		
20	3.9	0.66	7.9	0.010	0.41	0.0031	1,768	-4.6	-4.91	-12.08	-1.25		
22	4.3	0.73	8.3	0.028	0.59	0.0004	1,821	-3.6	-4.11	-12.63	-1.22		
24	7.7	0.85		0.036	0.59	0.021	1,899	-3.6	-4.25	-11.85	-1.31		
26	13	0.62	8.5	0.041	0.52	0.0022	2,121	-2.9	-3.61	-12.12	-1.14		
28	16	0.82		0.036	0.45	0.0024	2,297	-1.9	-3.66	-12.57	-1.19		
30	22	0.51	13	0.034	0.42	0.0011	2,445	-4.2	-3.51	-13.05	-1.07		
32	25	0.60	13	0.031	0.40	0.0010	2,565	-1.4	-4.85	-12.01	-1.26		
34	36	0.45	18	0.055	0.41	0.0038	2,805	-2.2	-5.08	-11.53	-1.11		

The values of δ^13^C-TDIC were from -5.08‰ to -2.78‰ vs. V-PDB, whereas those of δ^18^O-H_2_O and δD-H_2_O ranged from -13.1‰ to -11.5‰ and from -1.4‰ to -1.1‰ vs. V-SMOW, respectively.

### Chemical and isotopic composition of dissolved gases

The chemical (in atm) and isotopic (δ^13^C-CO_2_, δ^13^C-CH_4_ and δD-CH_4_) composition of dissolved gases is reported in Table 2 and Fig 3C. Atmospheric-related gases (N_2_, O_2_ and Ar) largely dominated at depth ≤12 m, their sum ranging from 0.60 to 0.90 atm. Deeper waters were anoxic and showed increasing amounts of CO_2_, CH_4_, H_2_ and He (up to 0.093, 0.64, 0.013 and 0.000095 atm, respectively) down to the maximum depth, where the total gas pressure was 1.33 atm. The measured TGP (Total Gas Pressure) was in the range of that measured in 2005 [26] but significantly higher with respect to that obtained in 2010 [31]. The δ^13^C-CO_2_ values ranged from -13.2‰ and -8.2‰ vs. V-PDB. The δ^13^C-CH_4_ values ranged from -65.4‰ to -50.7‰ vs. V-PDB, whereas δD-CH_4_ varied from -275‰ to -267‰ vs. V-SMOW.

**Table 2:** Sampling depth (m) and chemical composition (in atm) of the main dissolved gases in samples from Lake Averno. The isotopic composition of dissolved CO_2_ (δ^13^C-CO_2,_ in ‰ vs. V-PDB) and CH_4_ (δ^13^C-CH_4_ and δD-CH_4_, in ‰ vs. V-PDB and ‰ vs. V-SMOW, respectively), and the *P*CO_2_ values expected at equilibrium with alkalinity (*P*CO_2calc_, in atm) are also reported.

depth	*P*CO_2_	*P*N_2_	*P*Ar	*P*CH_4_	*P*O_2_	*P*H_2_	*P*He	pTOT	δ^13^C(CO_2_)	δ^13^C(CH_4_)	δD(CH_4_)	*P*CO_2calc_
0	0.000026	0.76	0.0090		0.14		0.000005	0.90				0.00018
2	0.000030	0.74	0.0085		0.14		0.000005	0.88				0.00018
4	0.000082	0.72	0.0085		0.16		0.000006	0.88				0.00027
6	0.00013	0.62	0.0073		0.20		0.000008	0.83	-11.9			0.0014
8	0.00028	0.60	0.0069		0.22		0.000005	0.82	-12.3			0.0077
10	0.00070	0.57	0.0066	0.000026	0.13		0.000008	0.71	-13.2	-50.7		0.0080
12	0.0014	0.57	0.0065	0.00029	0.029		0.000010	0.60	-12.8	-53.1	-267	0.0080
14	0.0047	0.57	0.0066	0.00073		0.00023	0.000007	0.58	-12.0	-53.6	-271	0.0083
16	0.0048	0.56	0.0065	0.0016		0.00058	0.000015	0.57	-12.2	-54.1	-269	0.0086
18	0.0075	0.56	0.0063	0.0045		0.00092	0.000020	0.69	-10.6	-54.7	-272	0.011
20	0.010	0.57	0.0065	0.024		0.0013	0.000020	0.67	-13.3	-56.9	-275	0.015
22	0.015	0.56	0.0064	0.052		0.0022	0.000027	0.64	-12.1	-58.1	-270	0.022
24	0.025	0.57	0.0063	0.10		0.0033	0.000037	0.70	-11.5	-59.5	-269	0.034
26	0.030	0.57	0.0065	0.19		0.0054	0.000052	0.80	-10.9	-62.2	-275	0.055
28	0.053	0.58	0.0063	0.30		0.0073	0.000062	0.94	-9.2	-63.4	-272	0.079
30	0.065	0.58	0.0062	0.47		0.0075	0.000065	1.12	-11.3	-65.2	-268	0.100
32	0.085	0.58	0.0062	0.50		0.010	0.000077	1.18	-8.2	-65.1	-271	0.13
34	0.093	0.59	0.0061	0.64		0.013	0.000095	1.33	-9.1	-65.4	-269	0.21

### Bacterial and archaea abundance and diversity

**Fig 4:**
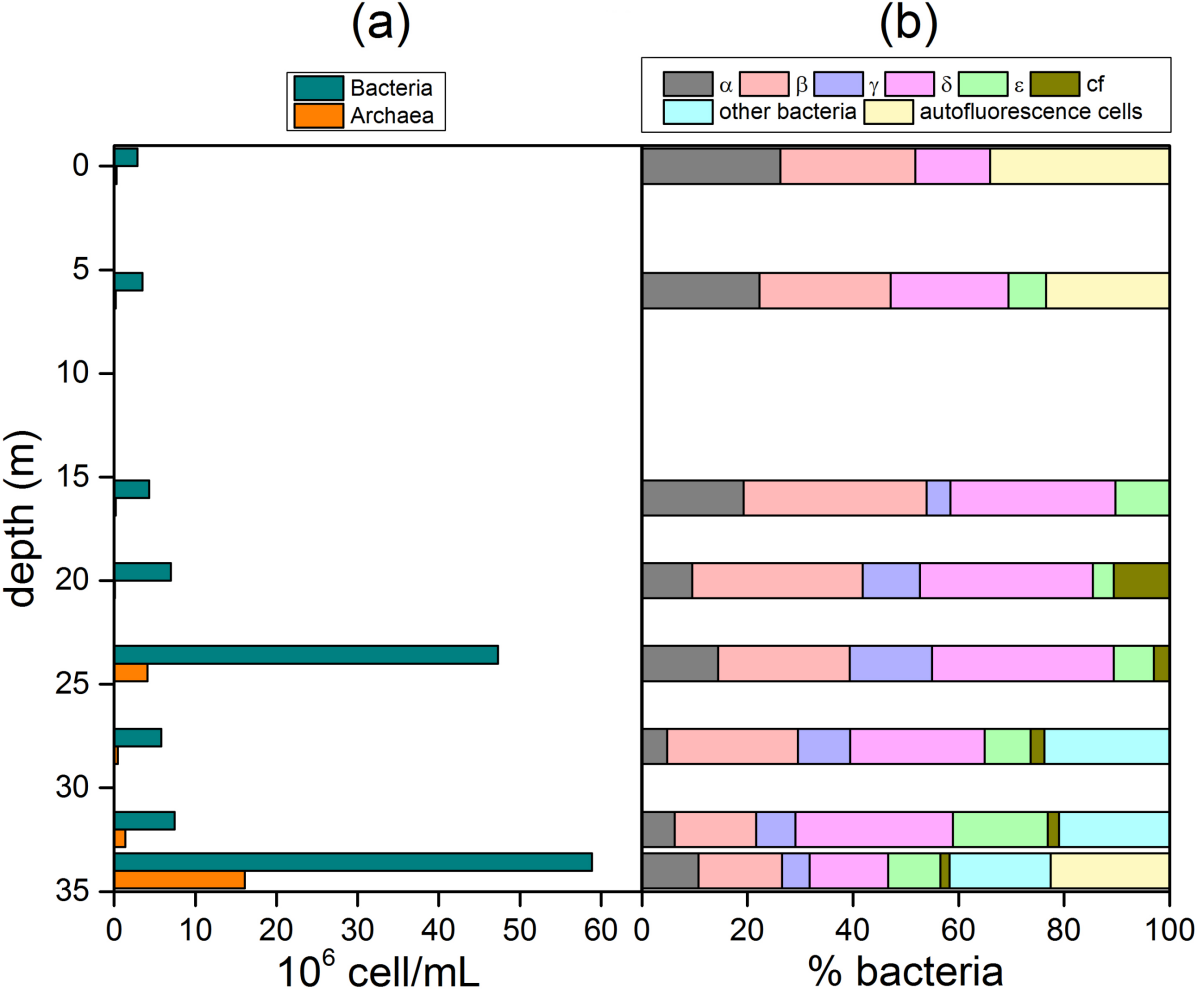
Vertical microbiological profiles along the Lake Averno water column estimated by CARD-FISH. (a) archaea and bacteria (cell ml^-1^); (b) Proteobacteria (Alpha, Beta, Gamma, Delta and Epsilonproteobacteria), *Bacteroidetes*-Flavobacteria (cf) and autofluorescent cells (expressed as % of total bacteria).

The average prokaryotic abundance, analyzed by epifluorescence microscopy, showed the lowest values at 0–16 m depth, the highest values being recorded at 24 m depth (7.3×10^7^ ± 1.72×10^7^ cell/mL) and at the lake bottom (1.01×10^8^ ± 9.8×10^6^ cell/mL at 34 m depth). Overall, Bacteria (probe EUB338 I-III) represented about 60% of total DAPI stained cells, whereas Archaea (probe ARCH 915) ranged from 0.9% to 16%, with the highest abundances at 24 m and 34 m depth (4.1×10^6^ ± 9.7×10^5^ cell/mL and 1.6×10^7^ ± 1.6×10^6^ respectively) (Fig 4A). Most Bacteria were affiliated to the phylum of Proteobacteria (79.51% ± 15.05). Among them, Alphaproteobacteria showed a decreasing trend with depth, from 26% at the lake surface to 11% at the lake bottom (percentages refer to total bacteria determined by CARD-FISH). Similarly, Betaproteobacteria ranged from 26 to 16%. Both Gammaproteobacteria and Deltaproteobacteria showed the highest percentages at 24 m depth (16% and 34%, respectively). Epsilonproteobacteria showed the highest percentage (18%) at 32 m depth. Bacteroidetes-Flavobacteria, not detected at 0–16 m depth, showed the highest percentage (11%) at 20 m depth, whereas other Bacteria were up to 24%, 21% 19% at 28, 32, and 34 m depth respectively (Fig 4B). Autofluorescence cells represented about 34% and 23% of bacterial cells at 0 and 6 m depth, respectively (10% filaments and 90% rod). At the lake bottom, the percentage of the autofluorescence cells was 23% (100% rod shape).

NGS analysis retrieved a total of 198 OTUs (see S1 Fig in supplementary material): 182 affiliated to 22 known phyla and 16 OTUs to unknown phyla. When the data is normalized to the same number of reads (subsample of 1,500 reads) they distributed along the vertical water column, as follows: 43 (0 m), 41 (-6 m), 47 (-16 m), 78 (-20 m), 105 (-24 m), 126 (-28 m), 124 (- 32 m), 128 (-34 m) OTUs. Shannon index showed an increasing trend with depth [2.6 (0 m), 2.3 (-6 m), 2.7 (-16 m), 2.9 (-20 m), 3.3 (-24 m), 3.8 (-28 m), 3.7 (- 32 m), 3.6 (-34 m)].

**Fig 5:**
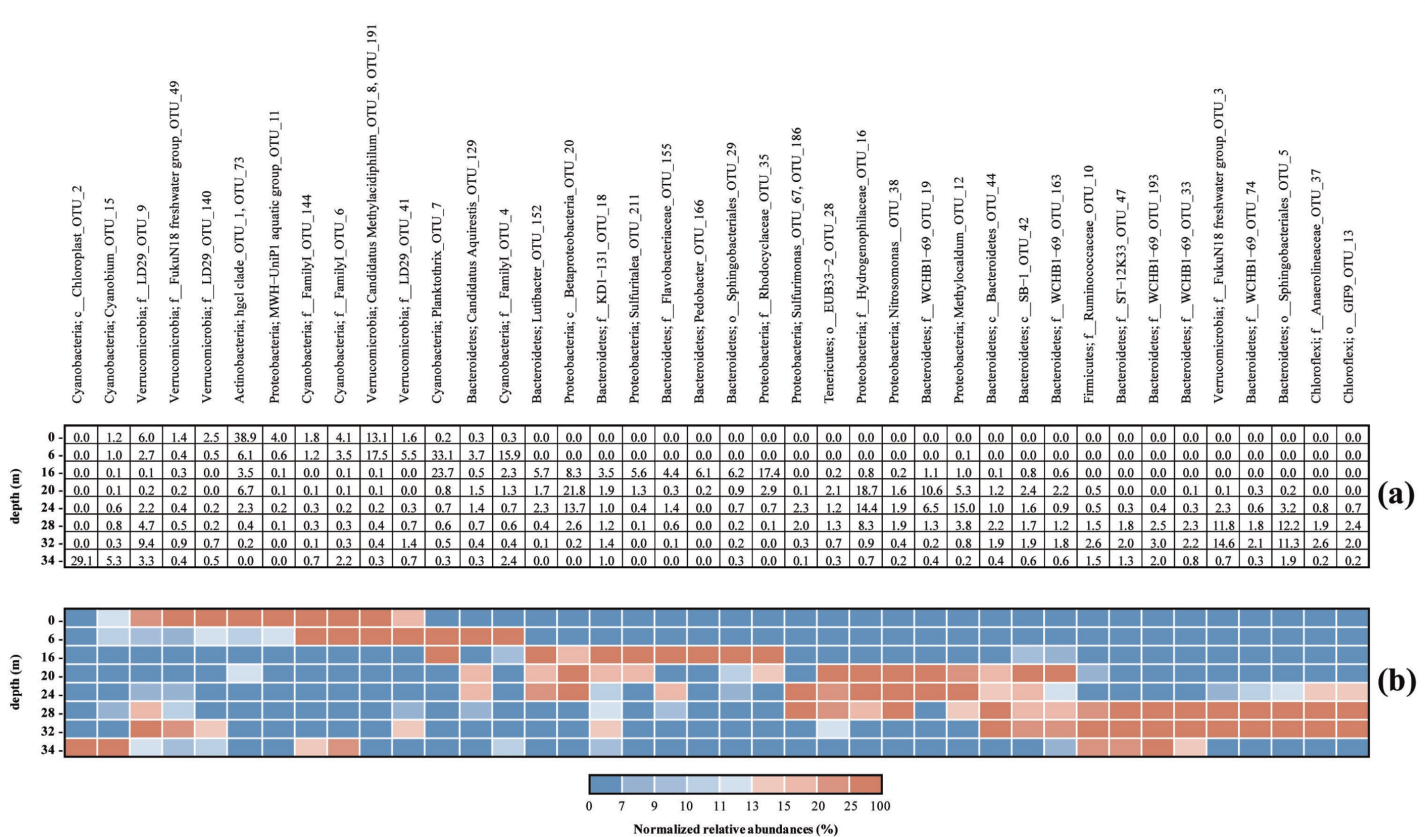
Relative abundance of the 40 most abundant bacterial genera along the Lake Averno water column. (A) Data are normalized to percent of total OTUs. (B) Table cells are colored (gradient scale from blue to red) according to the relative abundances. The abundance of each genus was normalized with respect to the average abundance. Hence, this allowed visualization of the relative increase or decrease in abundance of each genus at the 8 sampling depths, despite the differences in abundance between genera. In addition, the genera were clustered (the y-axis) to visualize by different shades of color those with similar patterns. Each group have both a broad group name (Phylum) and a specific name (Genus). If no genus name could be assigned, the best assignment is reported.

At the lake surface, Actinobacteria OTUs were found at the highest percentages (38.9% of total OTUs). At 6 m depth, i.e. within the photic zone, the analysis showed the dominance of photosynthetic taxa and aerobic microbial functional groups including putative methane-oxidizing bacteria related to *Verrucomicrobia* (e.g. *Candidatus* Methylacidiphilum spp.; 17.5%) that were also relatively abundant at the lake surface (13.1%). The relative abundance of 16S rRNA gene sequences belonging to phototrophic prokaryotes accounted for 54.7%. In particular, cyanobacterial *Planktothrix* sequences were retrieved at high percentage (33.1%). Sequences related to *Aquirestis* species within Bacteroidetes phylum were also found (3.7%) (Fig 5).

At 16 m depth, i.e. where no free O_2_ was detected, a variety of anaerobic microbial functional groups were recognized. Facultative anaerobic microorganisms affiliated to Rhodocyclaceae (Betaproteobacteria) were found at high abundance (17.4%). Additionally, other Betaproteobacteria were affiliated to Sulfuritalea (5.6%). OTUs related to Bacteroidetes phylum (32.6%) were affiliated to a variety of genera, such as Pedobacter (6.1%) and Lutibacter (5.7%) and Sphingobacteriales (6.2%) (Fig 5).

At 20–24 m depth, a clear shift in terms of microbial community composition and abundance was observed. The most abundant 16S rRNA gene sequences were mainly affiliated to Hydrogenophilaceae (18.7% and 14.4% at 20 m and 24 m depth, respectively), *Methylocaldum* (15% at 24 m depth), and the unknown Betaproteobacterium OTU20 (21.8% and 13.7% at 20 m and 24 m depth, respectively) (Fig 5). Remarkably, in line with CARD-FISH data that revealed the occurrence of Archaea from 24 m to 35 m depth (Fig 4A), the 5 Archaea OTUs retrieved, all belonging to the family Deep Sea Hydrothermal Vent Gp 6 (DHVEG-6), were found with an increasing relative abundance below—24 m depth (0.02%, 0.38%, 0.63%, 0.71% at 24 m, 28 m, 32 m, and 35 m depth, respectively).

At depth >28 m, the microbiome was composed by several anaerobic genera related to acidogens (Firmicutes Bacteroidetes, Sphingobacterales) and to anaerobic and chemoorganotrophic *Anaerolinea* [64]. Members of Spartobacteria class (Verrumicrobia) (27.8%) and chloroplast associated sequences (up to 29.6% at the lake bottom) were also found. OTUs affiliated to picocyanobacteria *Cyanobium* were also retrieved at the deepest water layer (5.3%).

**Fig 6:**
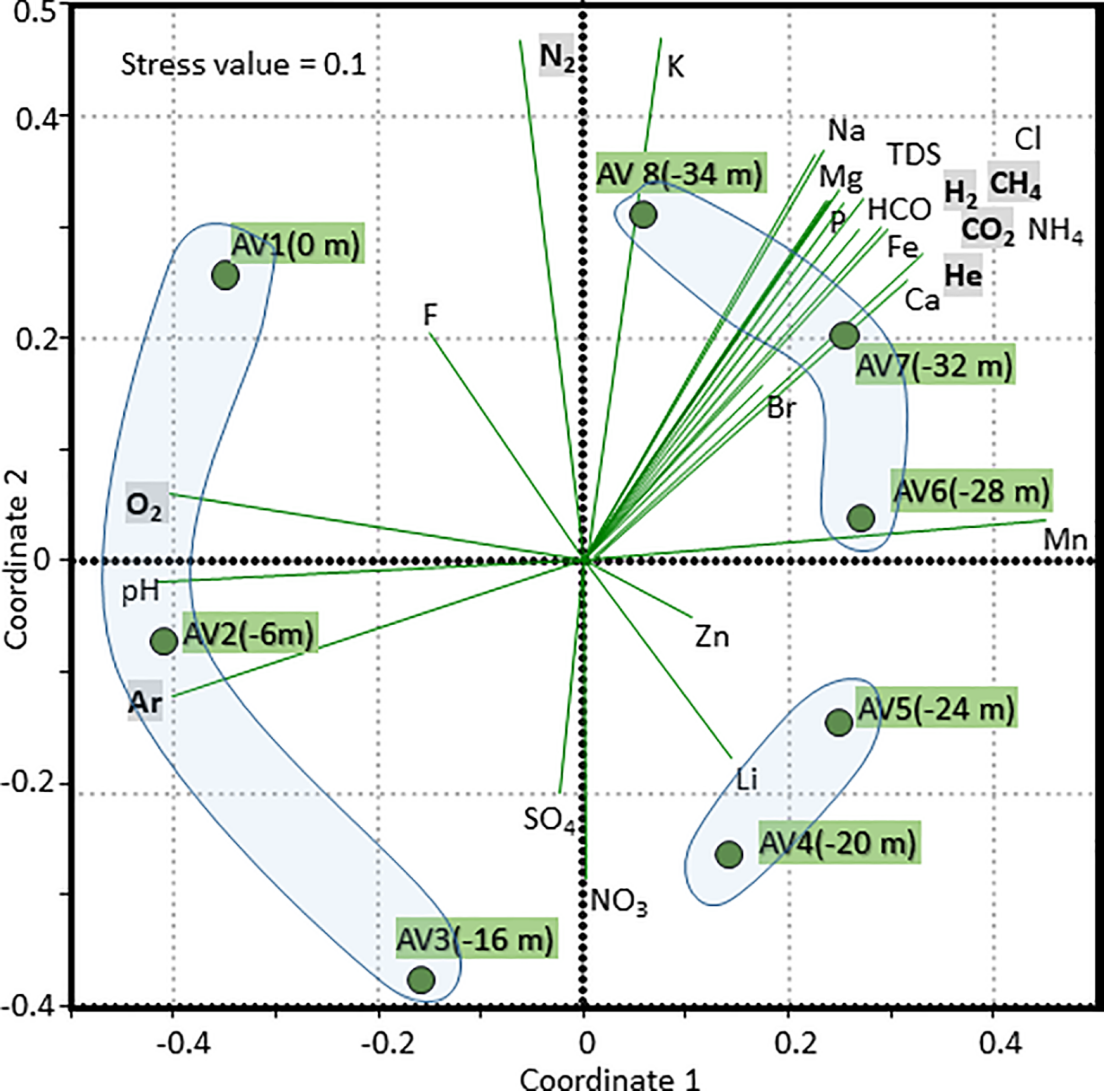
Relationships between environmental variables (chemical parameters and dissolved gases) and taxonomic composition, considering the 40 most abundant bacterial genera in the samples across sites. Nonmetric MultiDimensional Scaling (NMDS) ordination plot represents the typifying microbial composition in the transition from the surface to deep waters. Stress value indicates the significant concordance between the distance among samples in the NMDS plot and the actual Bray-Curtis distance among samples. Each dot represents the microbial community at a specific depth. Distance between the sample dots signifies similarity; the closer the samples are, the more similar microbial composition they have. The chemical parameters were incorporated in the NMDS analysis with a vector-fitting procedure.

Overall, as shown by the Nonmetric MultiDimensional Scaling (NMDS) ordination plot, the prokaryotic biodiversity of Lake Averno analyzed by NGS revealed a vertical stratification of the major microbial groups and a close relation to the gradient of the physicochemical parameters. In particular, the most important explaining factors were O_2_, Ar, pH and Mn, that showed a significant correlation (P<0.05) with NMDS Axis 1, that clearly discriminated among superficial and deep waters (Fig 6).

## Discussion

### Water and dissolved gas sources

The chemistry of volcanic lakes basically depends on the mass balance between fluids from the hydrothermal-magmatic system and meteoric water [4]. At Lake Averno, which has an elliptical surface of 0.54 km^2^ and a volume of ~6×10^6^ m^3^ the occurrence of a 1 km long and ~2 m wide outlet with a constant average flow of ~40 L/s suggests that sub-lacustrine springs, likely having chemical features similar to the hydrothermal emergences characterizing the Campi Flegrei [65], are present, although they were not directly observed. Hence, it is not surprising that clear clues of hydrothermal-magmatic inputs were recognized [26], as follows: 1) a Na^+^-Cl^-^ composition, similar to that of fluids exploited from deep wells drilled in this area for geothermal prospection [66]; 2) relatively high SO_4_^2-^ concentrations (Table 1), interpreted as due to dissolution of H_2_S from the hydrothermal system; 3) δ^18^O-H_2_O and δD-H_2_O values (Table 1) isotopically heavier than those of the local meteoric water (-6‰ and -35‰ vs. V-SMOW, respectively) [67], which were considered as related to deep water contribution; 4) high He/Ar ratios (up to 0.0034), which were up to 1 order of magnitude higher than those of air saturated water (ASW) at 10–26°C. However, a simple mixing process between primary sources (meteoric and hydrothermal) cannot completely explain the chemical features shown by Lake Averno. The carbon isotopic signature of CO_2_ (Table 2), which is the main gas constituent of hydrothermal fluids, was more negative than that measured in deep-originated gases discharged from the Campi Flegrei area (~-1.4‰ vs. V-PDB), and significantly more negative than those characterizing other volcanic lakes from central-southern Italy [31,36]. Moreover, notwithstanding the strong relationship between *P*CO_2_ and pH at depth <16 m (Tables 1 and 2), the *P*CO_2_ values were significantly lower than those expected at equilibrium with alkalinity (*P*CO_2calc_; Table 2) at the measured pH, salinity and temperature [68]. The disagreement between the measured and computed *P*CO_2_ values, coupled with the negative δ^13^C-CO_2_ values, clearly indicates that CO_2_ addition from the hydrothermal system was not the only process governing the behavior of this gas compound at Lake Averno. This evidence is supported by the relatively high *P*CH_4_ values, which cannot be ascribed to hydrothermal gas inputs for two main reasons: 1) hydrothermal fluid discharges at Campi Flegrei have CH_4_/CO_2_ ratios [69] orders of magnitude lower than those measured in the lake; 2) the δ^13^C-CH_4_ and δD-CH_4_ values measured at Lake Averno (Table 2) are typical for CH_4_ produced by microbial activity, thus not consistent with the environmental conditions of a hydrothermal reservoir. In fact, the carbon isotopic signature of dissolved CH_4_ (from -50‰ to -65.4‰ vs. V-PDB; Table 1) was significantly more negative with respect to that of the typical thermogenic gases (>-40‰ vs. V-PDB) [47,70]. A significant contribution to the Lake Averno water from a pure organic source having strongly negative δ^13^C (-25‰ vs. V-PDB; [71]) is also confirmed by the δ^13^C-TDIC values, which showed relatively low negative values to be related to volcanic/hydrothermal fluids [26]. Hence, microbial activity in lake water and, likely, within the sediments of the lake bottom seems to have a dominant control on the C-bearing chemical species of this volcanic lake, although the main solutes are provided by the hydrothermal system. Similar considerations are likely valid for other dissolved gases (e.g. O_2_ and H_2_) and ions (e.g. S- and N-bearing compounds, P, Fe_tot_, Mn and Zn), commonly involved in biogeochemical processes.

### Vertical profiles of lake chemistry vs. prokaryotic activity

At the lake surface, 16S rRNA gene sequences affiliated to Cyanobacteria and high relative abundances of Actinobacteria OTUs were retrieved. Even though Actinobacteria are considered typical inhabitants of soil environments, several studies reported this class as common also in a variety of freshwater habitats [72–73]. Owing to the small numbers of currently existing isolates of limnetic Actinobacteria, very little is known about their metabolic traits. Nevertheless, several studies reported the capability of Actinobacteria to grow at high pH, temperature and water stress conditions [74]. A distinguishable feature of this group is related to their capability to utilize a variety of substrates including the less degradable ones like chitin, cellulose and hemicellulose and to be resistant to UV radiation [72]. Overall, these peculiarities may explain their occurrence in photic zone of the lake, where complex carbon substrates can also be available. Hence, biological CO_2_ consumption and the relatively high pH (Fig 2B), causing a rapid CO_2_ dissolution to form HCO_3_^-^ and CO_3_^2-^, explain the extremely low CO_2_ concentrations measured in this water layer (Table 2).

At ~6 m depth, i.e. where water is still lighted and oxygenated, typical phototrophic prokaryotes and metanotrophs, e.g. *Candidatus* Methylacidiphilum affiliated to Verrucomicrobia, were found, consistent with the relatively low CH_4_ concentrations at 6–15 m depth (Fig 3C). It is worth to notice that methanotrophic Verrucomicrobia were previously reported in geothermal environments at low pH values. The presence in the Averno lake surface waters, characterised by high pH, is in line with the recent hypothesis that these bacteria could be present under a broad range of environmental conditions [75]. Similar results were found in other meromictic volcanic lakes, where CH_4_ oxidation, as well as CO_2_ consumption through oxygenic photosynthesis, was reported to occur in the epilimnion [76–77]. The presence of cyanobacterial filamentous microorganisms (*Planktothrix* spp.), often associated with summer massive blooms in freshwater lakes and reservoirs [78] and typically using CO_2_ as primary carbon source, strongly suggests the important role played by microorganisms on determining the CO_2_ vertical profile in the epilimnion. In this shallow water layer, *Aquirestis* species affiliated to Bacteroidetes phylum were also detected. They are bacteria frequently occurring in the pelagic zone of natural freshwater lakes and ponds with a limited number of cultured representatives [79]. Some *Aquirestis* species are restricted to hard-water habitats, characterized by a Ca^2+^(Mg^2+^)-HCO_3_^-^ composition and pH ≥7.7 [79].

At ~16 m depth, i.e. below the oxic-anoxic interface, a rich microbial diversity, with pronounced vertical structure in terms of taxonomic and potential functional composition, was found, mainly consisting of anoxygenic photoheterotrophs, denitrifiers, acidogens and sulfur-oxidizing chemoautotrophs as evidenced by the presence of cyanobacteria *Planktothrix*, Rhodocyclaceae and *Sulfuritalea* [80,81]. In particular, members of Sulfuritalea were recognized to be able to grow chemolithoautotrophically using ΣS_2_^-^ as electron donors and O-bearing ions, such as NO_3_^-^, as electron acceptors [82]. Overall, the metabolic potential highlighted by the 16S rRNA gene sequencing data are consistent with the significant increase of the ΣS^2-^/SO_4_^2-^ and NH_4_^+^/NO_3_^-^ ratios (Fig 3B).

At 20–24 m depth, a strong increase of the microbial abundance was observed. Both CARD-FISH and NGS showed the dominance of Deltaproteobacteria (Fig 3B), which were likely responsible for sulfate reduction [83], consistent with the pronounced increasing trend of the ΣS^2-^/SO_4_^2-^ ratio (Fig 3B) notwithstanding the contemporaneous occurrence of sulfur-oxidizing bacteria (*Sulfuritalea* and *Sulfurimonas*). High abundances of 16S rRNA gene sequences mainly affiliated to *Hydrogenophilaceae* were retrieved, likely related to ΣS^2-^ availability. Most members of this family are indeed mixotrophic or chemolithotrophic, being able to use various reduced sulfur compounds or hydrogen as electron donor [84]. The significant increase of dissolved H_2_ concentrations at increasing depth (Table 2) was likely due to the presence of H_2_-producing purple non-sulfur bacteria [85]. It is worth noting that 16S rRNA gene sequences of aerobic CH_4_ oxidizing bacteria *Methylocaldum* spp. were found. Members of this genus are commonly reported to grow aerobically, their occurrence in the Lake Averno hypolimnion was unexpected, although little is known about the metabolic potential of this functional group, which was found in a variety of environments, such as marine sediments [86] and engineered systems [87]. Recently, a methanotrophic strain, named *Methylocaldum* sp. SAD2, was isolated from an H_2_S-rich anaerobic digester [88]. It grew stably on CH_4_/air mixtures containing 500 and 1,000 ppm of H_2_S, and showed H_2_S tolerance higher than that reported for other known methanotrophs such as *Methylomicrobium* spp. and *Methylocystis* spp. These findings suggest that *Methylocaldum* spp. likely contributed to CH_4_ consumption even at anaerobic conditions, as observed in other meromictic lakes [89]. This intriguing hypothesis needs to be further investigated, since it may open new insights on the metabolic potentialities of such microbes and on the overall role of these environments in reducing CH_4_ emission. It is worth to note that CH_4_ oxidation in anoxic lake waters was recently reported to be carried out by aerobic gammaproteobacterial methanotrophs, able to respire electron acceptors other than oxygen [90–92]. Moreover, recent evidences showed the capability of *Candidatus* Methylomirabilis oxyfera (candidate division NC10) to anaerobically perform CH_4_ oxidation with O_2_ generated intracellularly by splitting reduced NO to N_2_ and O_2_ [93]. This is particularly of interest taking into account that so far the anaerobic CH_4_ oxidation has been ascribed exclusively to anaerobic methanotrophic archaea (ANME) alone or in synergistic relationship with bacteria [94–96].

The anaerobic CH_4_ consumption was likely counteracted by Archaea, whose presence in water was highlighted by CARD-FISH (Fig 4A). However, the vertical profiles of both the CH_4_ concentrations and the δ^13^C-CH_4_ values (Fig 3C) indicate predominance of CH_4_ consumption over production, as already observed in other volcanic lakes [31, 32]. This in line with NGS data showing that the few Archaea OTUs (up to 0.8%) belong to uncultivable Halobacteria, related to the family of the Deep Sea Hydrothermal Vent Gp 6 (DHVEG-6). This family was detected in different environments, such as seafloor methane seeps and hydrothermal fields [97–98], and associated with the availability of reduced inorganic sulphur compounds and slightly brackish waters [99]. Overall, the occurrence of multiple metabolic features, likely favored by the enhanced concentrations of electron acceptors (Fig 3B), might explain the relatively high prokariotic abundance detected at 24 m depth (Fig 4A).

At depths >28 m, several anaerobic genera related to known acidogens (Firmicutes Bacteroidetes, Sphingobacterales, Verrucomicrobia*)* and to anaerobic and chemoorganotrophic *Anaerolinea* were found. Among them, Spartobacteria class (Verrucomicrobia) is one of the most abundant bacterial lineages in soil and was recently found to be ubiquitous in aquatic environments [100, 101]. Some representatives of this class are strictly anaerobic, mesophilic and carbohydrate-fermenting bacteria.

The latter evidence is consistent with the expected occurrence of anaerobic methanotrophy at Lake Averno (Fig 3C). Moreover, the occurrence of Chloroplast and picocyanobacteria *Cyanobium* associated sequences, which were found only in this sample, and the presence of autofluorescent cells observed by microscopy analysis, was most likely derived from near-surface water.

**Fig 7:**
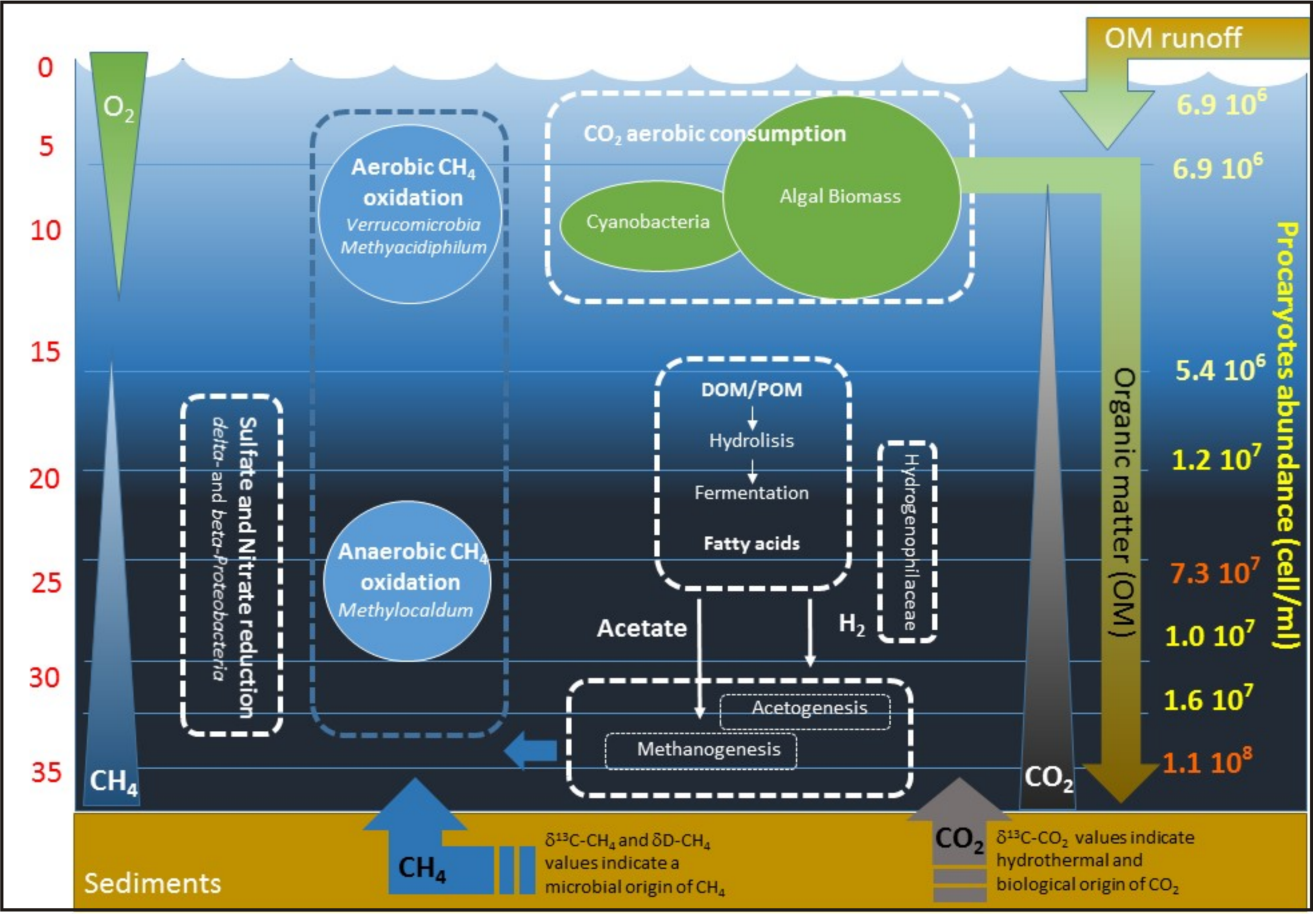
Schematic conceptual model of the interactions between microbial populations and geochemical parameters at different depth in Lake Averno.

Overall, at Lake Averno, the chemical, isotopic and microbiological data consistently indicate a strong inter-dependence between the chemical features of the dissolved gas reservoir and microbial niche differentiation along the vertical water column (Fig 7).

### Effects of bio-geochemical processes on lake stability

Considering the worldwide distribution of meromictic lakes in volcanic systems [3], the limnic eruption is to be regarded as a relatively frequent phenomenon. Notwithstanding, thirty years after the dramatic event occurred at Lake Nyos, there is no a general agreement about the initial cause(s) that provoked the lake destabilization. Firstly, buildup and release of gases accumulated in the hypolimnion of a meromictic lake were explained as due to gas injection from the lake bottom [102], whereas an external intervention (earthquake and/or landslide) was invoked as a destabilizing factor of CO_2_ stored from 150 to 210 m depth [103]. Secondly, internal waves generated by disturbances due to wind blowing in the vicinity of the lake [104] were supposed to be able to provoke a lake rollover [105], an idea that was supported by the results of laboratory experiments on the stability of stratified water tanks [106]. A third hypothesis suggested spontaneous nucleation and growth of gas bubbles, which became unstable and violently rose up producing a limnic eruption, likely due to oversaturation of deep lake layers related to gradual accumulation of hydrothermal-volcanic gases [107–110]. In stratified volcanic lakes (e.g. Lake Kivu and Lake Nyos; [111]), double-diffusion (DD), a process that spontaneously forms layers of high-density gradient surrounded by nearly homogeneous waters [112, 113], may cause convective mixing of the water column. Under a DD convection regime, layers telescoping together along the vertical lake profile are supposed to produce an explosive venting [114].

#### Computed vertical profile of the density gradient

Despite the fact that defining random and/or periodic triggers of a limnic eruption are still a challenge, the density gradient is undoubtedly the main controlling factor for the stability of a meromictic lake. Hence, the evaluation of the potential hazard related to lake rollover events has to be based on this parameter.

The density of an aqueous solution (ρ) depends on temperature (T), salinity (S) and dissolved gases, as described by the following equation:

$$\rho =\rho \left(T,S\right)\times \left(1+\beta_{CO2}\times CO_{2}+\beta_{CH4}\times CH_{4}\right)$$ (2)

where β_CO2_ (2.84×10^−4^ kg/g) and β_CH4_ (-1.25×10^−3^ kg/g) are the contraction coefficients of CO_2_ and CH_4_, respectively, as reported by McGinnis et al. [115]. A practical approach to compute ρ(T, S) was proposed by Moreira et al. [116], as follows:

$$\rho \left(T,S\right)=\rho_{W}\left(T\right)+K_{25}\left[\lambda_{0}+\lambda_{1}\times \left(T-25{}^{\circ}C\right)\right]$$ (3)

where ρ_W_ (T) is the density of pure water at the lake water temperature (T), which can be calculated according to Tanaka [117], whereas K_25_ is the electrical conductivity at 25°C that was computed using the algorithm implemented in the PHREEQC code [118]. The λ_0_ and λ_1_ values are, as follows:

$$\lambda_{0}=\left[\rho \left(25{}^{\circ}C,K_{25}\right)-\rho_{W}\;\left(25{}^{\circ}C\right)\right]/K_{25}$$ (4)

$$\lambda_{1}=\left\{\left[\rho \left(T,K_{25}\right)-\rho_{W}\left(T\right)\right]/K_{25}-\lambda_{0}\right\}/\left(T-25{}^{\circ}C\right)$$ (5)

The ρ(25°C, K_25_) and ρ(T, K_25_) values were calculated on the basis of the partial molar volumes of the lake water at the sampling depth [119].

**Fig 8:**
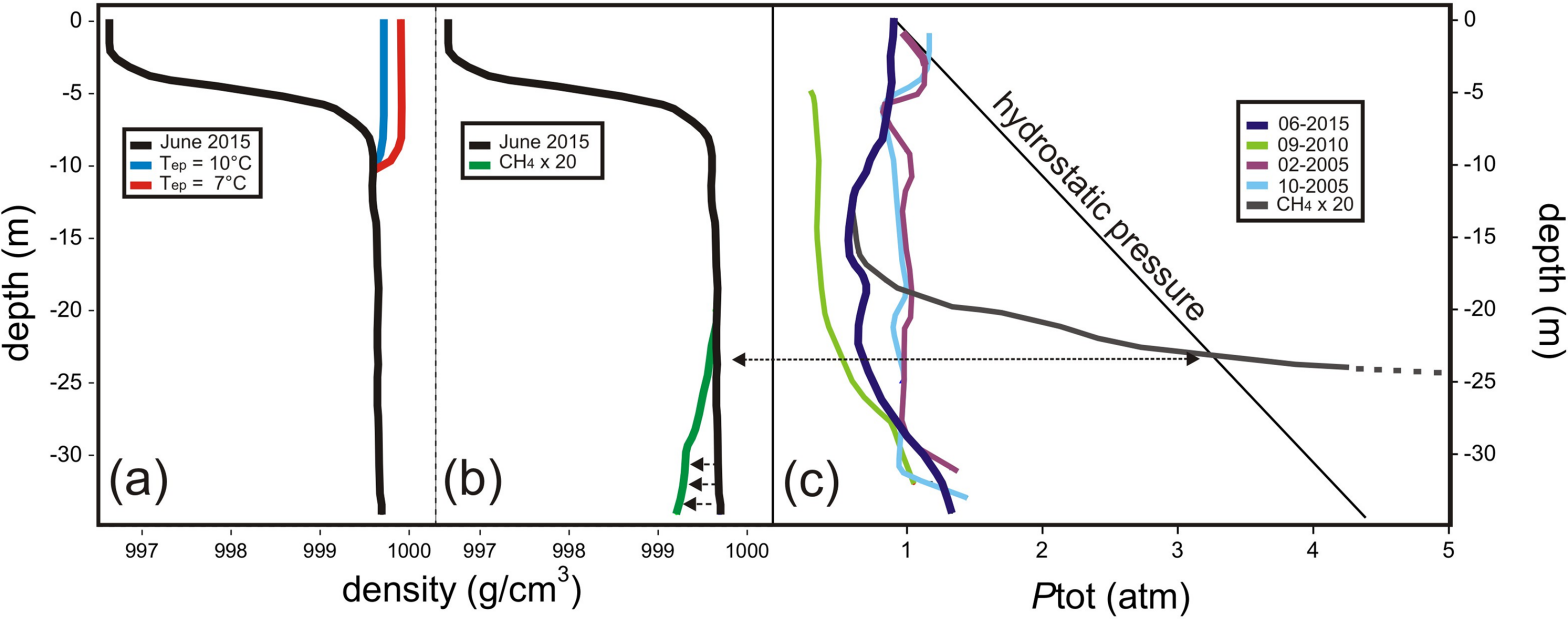
(a): Vertical profile of water density (in g/cm^3^), calculated using data measured in 2015 in Eq (2) (black line), compared to that computed assuming T = 7°C ([26]; red line) and T = 10°C (blue line) in the epilimnion. At the latter temperature, the density of epilimnetic water is higher than that measured at >8 m depth. (b): Vertical profile of water density (in g/cm^3^), calculated using data measured in 2015 in Eq (2) (black line), compared to that resulting from changes in the concentrations of dissolved CH_4_ and CO_2_ (considering CH_4_×20 and CO_2_ = 0; green line). The dashed arrows show the decrease of the water density at ≥20 m depth. (c): Vertical profiles of *P*tot of dissolved gases (in atm) in February and October 2005 ([26]; magenta and light blue lines, respectively), September 2010 ([31]; green line) and June 2015 (this paper, black line), compared with that of the hydrostatic pressure (black straight line). The vertical profile of *P*tot of dissolved gases in June 2015 assuming *P*CH_4_×20 (see Fig 8B) is also reported (grey line).

As shown in Fig 8A, the strong density gradient (black line) produced by both the thermocline and the chemocline confirms that on the 17th June 2015, when the sampling and measurement fieldtrip was carried out, Lake Averno was characterized by a stable stratification. According to this new dataset, when the temperature in the epilimnion (T_ep_) drops down to 10°C (blue line), epilimnetic waters (0–8 m depth) are denser than those at 8–26 m depth. At these conditions, the lake stratification is unstable. This T_ep_ limit value is significantly higher with respect to that (7°C; red line) resulting from the theoretical calculations carried out by Caliro et al. [26], suggesting that a lake rollover may occur when weather conditions, which control T_ep_, are less extreme than previously estimated.

#### Effect of dissolved gas chemical composition on the density gradient

As clearly shown by the values of β_CO2_ and β_CH4_ [113], dissolved CO_2_ increases water density, whereas dissolved CH_4_ (as well as other low-solubility gases such as H_2_ and N_2_) has an opposite effect. Hence, changes in the CH_4_ and CO_2_ concentrations affect the lake water density profile. It is worth noting that strong variations of the dissolved CH_4_ and CO_2_ concentrations have occurred in the last decade, corresponding to CH_4_/CO_2_ ratios at the lake bottom passing from ~1, in 2005–2006 [26,33], to 0.2 in 2010 [31] and 2015 (this paper). To evaluate the effect of the lake stratification potentially caused by changes in the chemical composition of the dissolved gas reservoir at Lake Averno, we hypothesized a strong increase of the measured CH_4_ concentrations (×20), in order to produce a significant decrease of the ρ values (up to ~0.5 g/L) in the deepest water layers (Fig 8B). Such a hypothetical CH_4_ increase, which is one order of magnitude higher with respect to the compositional variations observed in the last years, cannot occur since the corresponding *P*tot values (up to 13 atm) are strongly higher than the hydrostatic pressure (Fig 8C), the latter representing the limit value for dissolved gases. In other words, salinity and temperature are by far the most important parameters controlling the water density gradient at Lake Averno, whereas the dissolved gases play a secondary role. On the other hand, an external event, such as a sudden increase of the input rate of deep-originated gases though the lake bottom related to the activity of the magmatic-hydrothermal system, may directly cause an increase of the *P*tot values in the hypolimnion (e.g. ~3 times the values measured in 2015; Fig 8C) up to the saturation level. At these conditions, gas bubble nucleation and coalescence may occur [107] giving rise to a highly unstable bi-phase system prone to a gas outburst. In this case, this phenomenon, being triggered by the discharge rate of hydrothermal fluids, should be defined as a phreatic event instead of a limnic eruption.

## Conclusions

The occurrence of dissolved gas reservoirs is a common feature of meromictic volcanic lakes, especially those hosted within recently active craters that typically show funnel-shape morphologies and receive hydrothermal-magmatic gas contribution. Microbial communities able to develop under the physicochemical conditions of this peculiar environment have, at their turn, a strong influence on water and dissolved gas chemistry. Chemical reactions and prokaryotic populations controlling CO_2_ and CH_4_, i.e. the main constituents of the dissolved gas reservoir, are of particular interest, because the hazard related to rollover events is mainly associated with the behavior of these gases. The production and consumption of CO_2_ and CH_4_ act as main opposite pushing forces regulating the gradient of metabolic diversity in the anaerobic water layers. Compositional variations of the dissolved gas reservoir may theoretically decrease the water density of the hypolimnetic waters down to values comparable with that of the epilimnion only admitting an unreliable increase of the CH_4_ concentrations. Although temperature and salinity are the most important parameter controlling the vertical profile of the ρ values of Lake Averno, the prevailing CH_4_ microbial consumption occurring in the hypolimnion, indicated by the CH_4_ and δ^13^CH_4_ vertical profiles, suggests that the biological processes tend to stabilize the lake stratification.

However, oversaturation conditions in the hypolimnion cannot be excluded to occur during periods of volcanic unrest, when pulses of deep-originated gases may affect the lake. In this case, the gas outburst would be related to a volcanic event, a phenomenon that, by definition, is clearly distinguished with respect to a limnic eruption.

The consumption of CH_4_, started at anaerobic conditions, was completed in the epilimnion through aerobic oxidation. Carbon dioxide concentrations also definitely decreased due to the growth of photosynthetic biota (algal biomass and cyanobacteria), coupled with chemical dissolution at high pH values. Dead organic matter settled from these shallow layers to the lake bottom continuously fed the biogeochemical cycle described above. According to this schematic conceptual model, it is evident that the inputs of CO_2_ from the hydrothermal/magmatic system do not correspond to a comparable CO_2_ output from the lake surface, being this gas mostly used by microbiota and/or involved in physicochemical reactions within the lake. Overall, Lake Averno, and supposedly the numerous worldwide distributed volcanic lakes having similar features (namely bio-activity lakes), acts as a CO_2_ sink, displaying a significant influence on the local carbon budget.

## Supporting information

S1 Fig Operational taxonomic units (OTUs) relative abundance in water samples estimated by NGS. (PDF)
