# Supplementary material for: The biogeochemical vertical structure renders a meromictic volcanic lake a trap for geogenic CO2 (Lake Averno, Italy)
Source: PLoS One. 2018 Mar 6;13(3):e0193914. doi: 10.1371/journal.pone.0193914 (PMC5839588; doi:10.1371/journal.pone.0193914)
Supplement: S1 Fig — (PDF) [file pone.0193914.s001.pdf]

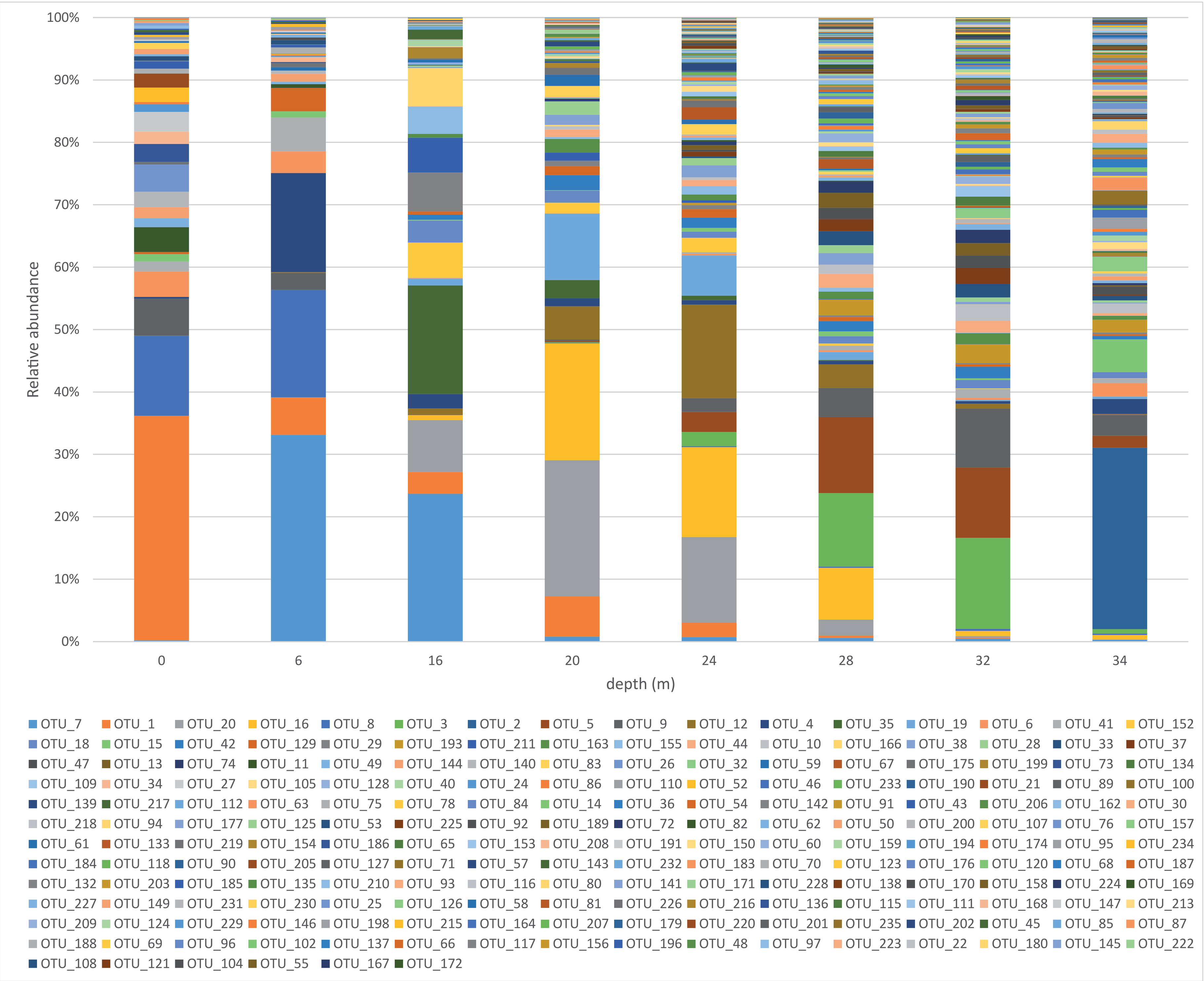

OTU	Kingdom	Phylum	Class	Order	Family	Genus	Species
OTU_1	k__Bacteria	p__Actinobacteria	c__Actinobacteria	o__Frankiales	f__Sporichthyaceae	g__hgcI clade	s__
OTU_2	k__Bacteria	p__Cyanobacteria	c__Chloroplast	o__	f__	g__	s__
OTU_3	k__Bacteria	p__Verrucomicrobia	c__Spartobacteria	o__Chthoniobacterales	f__FukuN18 freshwater group	g__	s__
OTU_4	k__Bacteria	p__Cyanobacteria	c__Cyanobacteria	o__SubsectionIII	f__FamilyI		
OTU_5	k__Bacteria	p__Bacteroidetes	c__Sphingobacteriia	o__Sphingobacteriales			
OTU_6	k__Bacteria	p__Cyanobacteria	c__Cyanobacteria	o__SubsectionI	f__FamilyI		
OTU_7	k__Bacteria	p__Cyanobacteria	c__Cyanobacteria	o__SubsectionIII	f__FamilyI	g__Planktothrix	s__
OTU_8	k__Bacteria	p__Verrucomicrobia	c__Verrucomicrobia Incertae Sedis	o__Unknown Order	f__Unknown Family	g__Candidatus Methylacidiphilum	s__
OTU_9	k__Bacteria	p__Verrucomicrobia	c__Spartobacteria	o__Chthoniobacterales	f__LD29	g__	s__
OTU_10	k__Bacteria	p__Firmicutes	c__Clostridia	o__Clostridiales	f__Ruminococcaceae	g__uncultured	s__
OTU_11	k__Bacteria	p__Proteobacteria	c__Betaproteobacteria	o__Burkholderiales	f__Alcaligenaceae	g__MWH-UniP1 aquatic group	s__
OTU_12	k__Bacteria	p__Proteobacteria	c__Gammaproteobacteria	o__Methylococcales	f__Methylococcaceae	g__Methylocaldum	s__
OTU_13	k__Bacteria	p__Chloroflexi	c__Dehalococcoidia	o__GIF9	f__	g__	s__
OTU_14	k__Bacteria	p__Chlorobi	c__Chlorobia	o__Chlorobiales	f__Chlorobiaceae	g__Chlorobium	s__
OTU_15	k__Bacteria	p__Cyanobacteria	c__Cyanobacteria	o__SubsectionI	f__FamilyI	g__Cyanobium	s__
OTU_16	k__Bacteria	p__Proteobacteria	c__Betaproteobacteria	o__Hydrogenophilales	f__Hydrogenophilaceae	g__uncultured	s__
OTU_18	k__Bacteria	p__Bacteroidetes	c__Sphingobacteriia	o__Sphingobacteriales	f__KD1-131	g__	s__
OTU_19	k__Bacteria	p__Bacteroidetes	c__Sphingobacteriia	o__Sphingobacteriales	f__WCHB1-69	g__	s__
OTU_20	k__Bacteria	p__Proteobacteria	c__Betaproteobacteria				
OTU_21	k__Bacteria	p__Cyanobacteria	c__Chloroplast	o__	f__	g__	s__
OTU_22	k__Bacteria	p__Proteobacteria	c__Alphaproteobacteria	o__Sphingomonadales	f__Sphingomonadaceae		
OTU_24	k__Bacteria	p__Bacteroidetes	c__Cytophagia	o__Cytophagales	f__Cyclobacteriaceae		
OTU_25	k__Bacteria	p__Cyanobacteria	c__Chloroplast	o__	f__	g__	s__
OTU_26	k__Bacteria	p__Bacteroidetes	c__Sphingobacteriia	o__Sphingobacteriales	f__Saprospiraceae	g__uncultured	s__
OTU_27	k__Bacteria	p__Bacteroidetes	c__Cytophagia	o__Cytophagales	f__Cyclobacteriaceae	g__	s__
OTU_28	k__Bacteria	p__Tenericutes	c__Mollicutes	o__EUB33-2	f__	g__	s__
OTU_29	k__Bacteria	p__Bacteroidetes	c__Sphingobacteriia	o__Sphingobacteriales			
OTU_30	k__Bacteria	p__Thermotogae	c__Thermotogae	o__Thermotogales	f__Thermotogaceae	g__SC103	s__
OTU_32	k__Bacteria						
OTU_33	k__Bacteria	p__Bacteroidetes	c__Sphingobacteriia	o__Sphingobacteriales	f__WCHB1-69	g__	s__
OTU_34	k__Bacteria	p__Proteobacteria	c__Alphaproteobacteria	o__Caulobacterales	f__Hyphomonadaceae	g__Hyphomonas	s__
OTU_35	k__Bacteria	p__Proteobacteria	c__Betaproteobacteria	o__Rhodocyclales	f__Rhodocyclaceae		
OTU_36	k__Bacteria	p__Bacteroidetes	c__vadinHA17	o__	f__	g__	s__
OTU_37	k__Bacteria	p__Chloroflexi	c__Anaerolineae	o__Anaerolineales	f__Anaerolineaceae	g__uncultured	s__
OTU_38	k__Bacteria	p__Proteobacteria	c__Betaproteobacteria	o__Nitrosomonadales	f__Nitrosomonadaceae	g__Nitrosomonas	s__
OTU_40	k__Bacteria	p__Bacteroidetes	c__Sphingobacteriia	o__Sphingobacteriales			
OTU_41	k__Bacteria	p__Verrucomicrobia	c__Spartobacteria	o__Chthoniobacterales	f__LD29	g__	s__
OTU_42	k__Bacteria	p__Bacteroidetes	c__SB-1	o__	f__	g__	s__
OTU_43	k__Bacteria	p__Proteobacteria	c__Alphaproteobacteria	o__Caulobacterales	f__Caulobacteraceae	g__Phenylobacterium	s__
OTU_44	k__Bacteria	p__Bacteroidetes					
OTU_45	k__Bacteria	p__Bacteroidetes	c__BSV13	o__	f__	g__	s__
OTU_46	k__Bacteria	p__Bacteroidetes	c__Bacteroidia	o__Bacteroidales	f__Porphyromonadaceae	g__Paludibacter	s__
OTU_47	k__Bacteria	p__Bacteroidetes	c__Sphingobacteriia	o__Sphingobacteriales	f__ST-12K33	g__	s__
OTU_48	k__Bacteria	p__Cyanobacteria	c__Chloroplast	o__	f__	g__	s__
OTU_49	k__Bacteria	p__Verrucomicrobia	c__Spartobacteria	o__Chthoniobacterales	f__FukuN18 freshwater group	g__	s__
OTU_50	k__Bacteria	p__Actinobacteria	c__Actinobacteria				
OTU_52	k__Bacteria	p__Actinobacteria	c__Actinobacteria	o__Micrococcales	f__Microbacteriaceae		
OTU_53	k__Bacteria	p__Bacteroidetes					
OTU_54	k__Bacteria						
OTU_55	k__Bacteria	p__Proteobacteria	c__Alphaproteobacteria	o__Rhizobiales	f__Phyllobacteriaceae	g__CD04	s__
OTU_57	k__Bacteria	p__Cyanobacteria	c__Chloroplast	o__	f__	g__	s__
OTU_58	k__Bacteria	p__Bacteroidetes	c__vadinHA17	o__	f__	g__	s__
OTU_59	k__Bacteria	p__Actinobacteria	c__Acidimicrobiia	o__Acidimicrobiales	f__Acidimicrobiaceae	g__	s__
OTU_60	k__Bacteria	p__Bacteroidetes	c__vadinHA17	o__	f__	g__	s__
OTU_61	k__Bacteria	p__Cyanobacteria	c__Chloroplast	o__	f__	g__	s__
OTU_62	k__Bacteria	p__Proteobacteria	c__Alphaproteobacteria	o__Sphingomonadales	f__Erythrobacteraceae	g__Porphyrobacter	s__
OTU_63	k__Bacteria	p__Chloroflexi	c__Dehalococcoidia	o__MSBL5	f__	g__	s__
OTU_65	k__Bacteria	p__Firmicutes	c__Clostridia	o__Clostridiales	f__Ruminococcaceae	g__Fastidiosipila	s__
OTU_66	k__Bacteria	p__Bacteroidetes	c__Sphingobacteriia	o__Sphingobacteriales	f__B01R012	g__	s__
OTU_67	k__Bacteria	p__Proteobacteria	c__Epsilonproteobacteria	o__Campylobacterales	f__Helicobacteraceae	g__Sulfurimonas	s__
OTU_68	k__Bacteria						
OTU_69	k__Bacteria						
OTU_70	k__Bacteria	p__Bacteroidetes	c__Bacteroidia	o__Bacteroidales	f__Marinilabiaceae	g__uncultured	s__
OTU_71	k__Bacteria	p__Proteobacteria	c__Deltaproteobacteria	o__Myxococcales	f__0319-6G20	g__	s__
OTU_72	k__Bacteria	p__Firmicutes	c__Clostridia	o__Clostridiales	f__Ruminococcaceae		
OTU_73	k__Bacteria	p__Actinobacteria	c__Actinobacteria	o__Frankiales	f__Sporichthyaceae	g__hgcI clade	s__
OTU_74	k__Bacteria	p__Bacteroidetes	c__Sphingobacteriia	o__Sphingobacteriales	f__WCHB1-69	g__	s__
OTU_75	k__Bacteria	p__Bacteroidetes	c__Sphingobacteriia	o__Sphingobacteriales	f__NS11-12 marine group	g__	s__
OTU_76	k__Bacteria	p__Bacteroidetes	c__WCHB1-32	o__	f__	g__	s__
OTU_78	k__Bacteria	p__Firmicutes	c__Erysipelotrichia	o__Erysipelotrichales	f__Erysipelotrichaceae	g__uncultured	s__
OTU_80	k__Bacteria	p__Firmicutes	c__Clostridia	o__Clostridiales	f__Ruminococcaceae		
OTU_81	k__Bacteria	p__Chloroflexi	c__Dehalococcoidia	o__GIF9	f__	g__	s__
OTU_82	k__Bacteria						
OTU_83	k__Bacteria	p__Bacteroidetes	c__Sphingobacteriia	o__Sphingobacteriales	f__B01R012	g__	s__
OTU_84	k__Bacteria	p__Bacteroidetes	c__Sphingobacteriia	o__Sphingobacteriales	f__WCHB1-69	g__	s__
OTU_85	k__Bacteria	p__Proteobacteria	c__Alphaproteobacteria	o__Rhizobiales	f__Nordellaceae	g__MNG7	s__
OTU_86	k__Bacteria	p__Bacteroidetes	c__Sphingobacteriia	o__Sphingobacteriales	f__LiUU-11-161	g__	s__
OTU_87	k__Bacteria						
OTU_89	k__Bacteria	p__Firmicutes	c__Erysipelotrichia	o__Erysipelotrichales	f__Erysipelotrichaceae	g__uncultured	s__
OTU_90	k__Bacteria	p__Chloroflexi	c__WCHB1-50	o__WCHB1-50	f__WCHB1-50	g__WCHB1-50	s__
OTU_91	k__Bacteria	p__Spirochaetae	c__Spirochaetes	o__LNR A2-18	f__	g__	s__
OTU_92	k__Bacteria	p__Bacteroidetes	c__Sphingobacteriia	o__Sphingobacteriales	f__Saprospiraceae	g__uncultured	s__
OTU_93	k__Bacteria	p__Firmicutes	c__Clostridia	o__Clostridiales	f__Ruminococcaceae	g__Incertae Sedis	s__
OTU_94	k__Bacteria	p__Chlamydiae	c__Chlamydiae	o__Chlamydiales	f__cvE6	g__	s__
OTU_95	k__Bacteria	p__Proteobacteria	c__Alphaproteobacteria	o__SAR11 clade	f__LD12 freshwater group	g__	s__
OTU_96	k__Bacteria	p__Chloroflexi	c__Anaerolineae	o__Anaerolineales	f__Anaerolineaceae	g__Anaerolinea	s__
OTU_97	k__Bacteria	p__Proteobacteria	c__Betaproteobacteria	o__Burkholderiales	f__Comamonadaceae		
OTU_100	k__Bacteria	p__Fibrobacteres	c__Fibrobacteria	o__Fibrobacterales	f__possible family 01	g__	s__
OTU_102	k__Bacteria						
OTU_104	k__Bacteria	p__Bacteroidetes	c__Sphingobacteriia	o__Sphingobacteriales	f__NS11-12 marine group	g__	s__
OTU_105	k__Bacteria	p__Proteobacteria	c__Betaproteobacteria	o__Methylophilales	f__Methylophilaceae	g__Methylotenera	s__
OTU_107	k__Bacteria	p__Proteobacteria	c__Alphaproteobacteria				
OTU_108	k__Bacteria	p__Bacteroidetes	c__WCHB1-32	o__	f__	g__	s__
OTU_109	k__Bacteria	p__Proteobacteria	c__Deltaproteobacteria	o__Syntrophobacterales	f__Syntrophaceae	g__Desulfobacca	s__
OTU_110	k__Bacteria	p__Bacteroidetes	c__vadinHA17	o__	f__	g__	s__
OTU_111	k__Archaea	p__Euryarchaeota	c__Halobacteria	o__Halobacteriales	f__Deep Sea Hydrothermal Vent Gp 6(DHVEG-6)	g__	s__
OTU_112	k__Bacteria	p__Bacteroidetes	c__SB-1	o__	f__	g__	s__
OTU_115	k__Bacteria						
OTU_116	k__Archaea	p__Euryarchaeota	c__Halobacteria	o__Halobacteriales	f__Deep Sea Hydrothermal Vent Gp 6(DHVEG-6)	g__	s__
OTU_117	k__Bacteria	p__Proteobacteria	c__Alphaproteobacteria	o__Rhizobiales	f__Nordellaceae	g__MNG7	s__
OTU_118	k__Bacteria	p__Candidate division TM7	c__	o__	f__	g__	s__
OTU_120	k__Bacteria	p__Firmicutes	c__Clostridia	o__Clostridiales	f__vadinBB60	g__	s__
OTU_121	k__Bacteria	p__Proteobacteria	c__Alphaproteobacteria				
OTU_123	k__Bacteria	p__Proteobacteria	c__Alphaproteobacteria	o__Rhodobacterales	f__Rhodobacteraceae	g__Gemmobacter	s__
OTU_124	k__Bacteria	p__Verrucomicrobia	c__Verrucomicrobiae	o__Verrucomicrobiales	f__Verrucomicrobiaceae		
OTU_125	k__Bacteria	p__Cyanobacteria	c__Melainabacteria	o__Gastranaerophilales	f__	g__	s__
OTU_126	k__Bacteria	p__Proteobacteria	c__Alphaproteobacteria	o__Rhizobiales	f__FukuN57	g__	s__
OTU_127	k__Bacteria	p__Chloroflexi	c__Anaerolineae	o__Anaerolineales	f__Anaerolineaceae	g__uncultured	s__
OTU_128	k__Bacteria	p__Verrucomicrobia	c__Opitutae	o__vadinHA64	f__	g__	s__
OTU_129	k__Bacteria	p__Bacteroidetes	c__Sphingobacteriia	o__Sphingobacteriales	f__Saprospiraceae	g__Candidatus Aquirestis	s__
OTU_132	k__Bacteria	p__Bacteroidetes	c__Bacteroidia	o__Bacteroidales	f__Porphyromonadaceae	g__Paludibacter	s__
OTU_133	k__Bacteria						
OTU_134	k__Bacteria	p__Proteobacteria	c__Deltaproteobacteria	o__Desulfarculales	f__Desulfarculaceae	g__uncultured	s__
OTU_135	k__Bacteria						
OTU_136	k__Bacteria	p__Bacteroidetes	c__Sphingobacteriia	o__Sphingobacteriales			
OTU_137	k__Bacteria	p__Verrucomicrobia	c__Opitutae	o__Opitutales	f__Opitutaceae	g__Opitutus	s__
OTU_138	k__Bacteria	p__Bacteroidetes	c__Sphingobacteriia	o__Sphingobacteriales	f__CMW-169	g__	s__
OTU_139	k__Bacteria	p__Verrucomicrobia	c__Verrucomicrobiae	o__Verrucomicrobiales	f__Verrucomicrobiaceae	g__uncultured	s__
OTU_140	k__Bacteria	p__Verrucomicrobia	c__Spartobacteria	o__Chthoniobacterales	f__LD29	g__	s__
OTU_141	k__Bacteria	p__Proteobacteria	c__Alphaproteobacteria				
OTU_142	k__Bacteria	p__Verrucomicrobia	c__Spartobacteria	o__Chthoniobacterales	f__FukuN18 freshwater group	g__	s__
OTU_143	k__Bacteria	p__Bacteroidetes	c__Flavobacteriia	o__Flavobacteriales	f__Cryomorphaceae	g__Fluviicola	s__
OTU_144	k__Bacteria	p__Cyanobacteria	c__Cyanobacteria	o__SubsectionI	f__FamilyI		
OTU_145	k__Bacteria	p__Proteobacteria	c__Alphaproteobacteria	o__Rhodobacterales	f__Rhodobacteraceae		
OTU_146	k__Bacteria	p__Cyanobacteria	c__Chloroplast	o__	f__	g__	s__
OTU_147	k__Bacteria	p__Firmicutes	c__Clostridia	o__Clostridiales	f__vadinBB60	g__	s__
OTU_149	k__Bacteria	p__Proteobacteria	c__Alphaproteobacteria	o__Caulobacterales	f__Hyphomonadaceae	g__Woodsholea	s__
OTU_150	k__Bacteria	p__Proteobacteria	c__Deltaproteobacteria	o__Syntrophobacterales	f__Syntrophaceae	g__Syntrophus	s__
OTU_152	k__Bacteria	p__Bacteroidetes	c__Flavobacteriia	o__Flavobacteriales	f__Flavobacteriaceae	g__Lutibacter	s__
OTU_153	k__Bacteria						
OTU_154	k__Bacteria						
OTU_155	k__Bacteria	p__Bacteroidetes	c__Flavobacteriia	o__Flavobacteriales	f__Flavobacteriaceae	g__uncultured	s__
OTU_156	k__Bacteria	p__Proteobacteria	c__Alphaproteobacteria	o__Rhizobiales	f__Hyphomicrobiaceae		
OTU_157	k__Bacteria	p__Chlorobi	c__Chlorobia	o__Chlorobiales	f__SJA-28	g__	s__
OTU_158	k__Bacteria	p__Bacteroidetes					
OTU_159	k__Bacteria	p__Candidate division OD1	c__	o__	f__	g__	s__
OTU_162	k__Bacteria	p__Proteobacteria	c__Gammaproteobacteria	o__Methylococcales	f__Methylococcaceae	g__Methylomonas	s__
OTU_163	k__Bacteria	p__Bacteroidetes	c__Sphingobacteriia	o__Sphingobacteriales	f__WCHB1-69	g__	s__
OTU_164	k__Archaea	p__Euryarchaeota	c__Halobacteria	o__Halobacteriales	f__Deep Sea Hydrothermal Vent Gp 6(DHVEG-6)	g__	s__
OTU_166	k__Bacteria	p__Bacteroidetes	c__Sphingobacteriia	o__Sphingobacteriales	f__Sphingobacteriaceae	g__Pedobacter	s__
OTU_167	k__Bacteria	p__Acidobacteria	c__Acidobacteria	o__Subgroup 4	f__Blastocatellaceae	g__AK1DE1	s__
OTU_168	k__Bacteria	p__Firmicutes	c__Clostridia	o__Clostridiales			
OTU_169	k__Bacteria						
OTU_170	k__Bacteria						
OTU_171	k__Archaea	p__Euryarchaeota	c__Halobacteria	o__Halobacteriales	f__Deep Sea Hydrothermal Vent Gp 6(DHVEG-6)	g__	s__
OTU_172	k__Bacteria	p__Verrucomicrobia	c__Verrucomicrobiae	o__Verrucomicrobiales	f__Verrucomicrobiaceae	g__Haloferula	s__
OTU_174	k__Bacteria	p__Bacteroidetes	c__Sphingobacteriia	o__Sphingobacteriales	f__NS11-12 marine group	g__	s__
OTU_175	k__Bacteria	p__Actinobacteria	c__Acidimicrobiia	o__Acidimicrobiales	f__Acidimicrobiaceae	g__	s__
OTU_176	k__Bacteria	p__Bacteroidetes	c__Bacteroidia	o__Bacteroidales	f__Porphyromonadaceae	g__Paludibacter	s__
OTU_177	k__Bacteria	p__Tenericutes	c__Mollicutes				
OTU_179	k__Bacteria	p__Proteobacteria	c__Betaproteobacteria	o__Burkholderiales	f__Comamonadaceae	g__Hydrogenophaga	s__
OTU_180	k__Bacteria	p__Proteobacteria	c__Alphaproteobacteria	o__Rhodobacterales	f__Rhodobacteraceae	g__Rubellimicrobium	s__
OTU_183	k__Bacteria	p__Bacteroidetes	c__Sphingobacteriia	o__Sphingobacteriales	f__	g__	s__
OTU_184	k__Bacteria	p__Synergistetes	c__Synergistia	o__Synergistales	f__Synergistaceae		
OTU_185	k__Bacteria	p__Bacteroidetes	c__Cytophagia	o__Cytophagales			
OTU_186	k__Bacteria	p__Proteobacteria	c__Epsilonproteobacteria	o__Campylobacterales	f__Helicobacteraceae	g__Sulfurimonas	s__
OTU_187	k__Bacteria	p__Bacteroidetes	c__SB-5	o__	f__	g__	s__
OTU_188	k__Bacteria	p__Bacteroidetes	c__Sphingobacteriia	o__Sphingobacteriales	f__Chitinophagaceae	g__uncultured	s__
OTU_189	k__Bacteria	p__Proteobacteria	c__Deltaproteobacteria	o__Desulfarculales	f__Desulfarculaceae	g__uncultured	s__
OTU_190	k__Bacteria	p__NPL-UPA2	c__	o__	f__	g__	s__
OTU_191	k__Bacteria	p__Verrucomicrobia	c__Verrucomicrobia Incertae Sedis	o__Unknown Order	f__Unknown Family	g__Candidatus Methylacidiphilum	s__
OTU_193	k__Bacteria	p__Bacteroidetes	c__Sphingobacteriia	o__Sphingobacteriales	f__WCHB1-69	g__	s__
OTU_194	k__Bacteria	p__Caldiserica	c__Caldisericia	o__Caldisericales	f__WCHB1-02	g__	s__
OTU_196	k__Bacteria	p__Firmicutes	c__Clostridia	o__Clostridiales	f__Ruminococcaceae		
OTU_198	k__Bacteria	p__Bacteroidetes					
OTU_199	k__Bacteria	p__Bacteroidetes	c__Flavobacteriia	o__Flavobacteriales	f__Cryomorphaceae	g__Fluviicola	s__
OTU_200	k__Bacteria	p__Caldiserica	c__Caldisericia	o__Caldisericales	f__WCHB1-02	g__	s__
OTU_201	k__Bacteria	p__Bacteroidetes	c__vadinHA17	o__	f__	g__	s__
OTU_202	k__Bacteria	p__Bacteroidetes	c__Sphingobacteriia	o__Sphingobacteriales	f__B01R012	g__	s__
OTU_203	k__Bacteria	p__Candidate division OD1	c__	o__	f__	g__	s__
OTU_205	k__Bacteria	p__Chlorobi	c__Ignavibacteria	o__Ignavibacteriales			
OTU_206	k__Bacteria	p__Bacteroidetes					
OTU_207	k__Bacteria	p__Candidate division OD1	c__	o__	f__	g__	s__
OTU_208	k__Bacteria	p__Bacteroidetes	c__SB-5	o__	f__	g__	s__
OTU_209	k__Bacteria	p__Candidate division OD1	c__	o__	f__	g__	s__
OTU_210	k__Bacteria	p__Proteobacteria	c__Gammaproteobacteria	o__Pseudomonadales	f__Moraxellaceae	g__Acinetobacter	s__
OTU_211	k__Bacteria	p__Proteobacteria	c__Betaproteobacteria	o__Rhodocyclales	f__Rhodocyclaceae	g__Sulfuritalea	s__
OTU_213	k__Bacteria	p__TM6	c__	o__	f__	g__	s__
OTU_215	k__Bacteria	p__Spirochaetae	c__Spirochaetes	o__Spirochaetales	f__Leptospiraceae	g__uncultured	s__
OTU_216	k__Bacteria						
OTU_217	k__Bacteria	p__Bacteroidetes					
OTU_218	k__Bacteria	p__Verrucomicrobia	c__OPB35 soil group	o__	f__	g__	s__
OTU_219	k__Bacteria	p__Spirochaetae	c__Spirochaetes	o__Spirochaetales	f__Spirochaetaceae	g__uncultured	s__
OTU_220	k__Bacteria	p__Proteobacteria	c__Gammaproteobacteria	o__Methylococcales			
OTU_222	k__Bacteria	p__Bacteroidetes	c__Sphingobacteriia	o__Sphingobacteriales	f__Saprospiraceae	g__CYCU-0281	s__
OTU_223	k__Bacteria	p__Chloroflexi	c__Anaerolineae	o__Anaerolineales	f__Anaerolineaceae		
OTU_224	k__Bacteria	p__Chloroflexi	c__Anaerolineae	o__Anaerolineales	f__Anaerolineaceae	g__Levilinea	s__
OTU_225	k__Bacteria	p__Actinobacteria	c__OPB41	o__	f__	g__	s__
OTU_226	k__Bacteria						
OTU_227	k__Bacteria	p__Cyanobacteria	c__Cyanobacteria	o__SubsectionI	f__FamilyI	g__Cyanobacterium	s__
OTU_228	k__Bacteria	p__Bacteroidetes					
OTU_229	k__Bacteria	p__BD1-5	c__	o__	f__	g__	s__
OTU_230	k__Bacteria	p__Candidate division OD1	c__	o__	f__	g__	s__
OTU_231	k__Bacteria	p__Cyanobacteria	c__ML635J-21	o__	f__	g__	s__
OTU_232	k__Bacteria	p__Bacteroidetes	c__Bacteroidia	o__Bacteroidales	f__Marinilabiaceae	g__uncultured	s__
OTU_233	k__Bacteria	p__Bacteroidetes	c__Sphingobacteriia	o__Sphingobacteriales			
OTU_234	k__Bacteria	p__Cyanobacteria	c__Chloroplast	o__	f__	g__	s__
OTU_235	k__Archaea	p__Euryarchaeota	c__Halobacteria	o__Halobacteriales	f__Deep Sea Hydrothermal Vent Gp 6(DHVEG-6)	g__	s__
